# Characterizing RNA Pseudouridylation by Convolutional Neural Networks

**DOI:** 10.1016/j.gpb.2019.11.015

**Published:** 2021-02-23

**Authors:** Xuan He, Sai Zhang, Yanqing Zhang, Zhixin Lei, Tao Jiang, Jianyang Zeng

**Affiliations:** 1 Institute for Interdisciplinary Information Sciences, Tsinghua University, Beijing 100084, China; 2 Peking-Tsinghua Center for Life Sciences, Peking University, Beijing 100871, China; 3 Academy for Advanced Interdisciplinary Studies, Peking University, Beijing 100871, China; 4 Department of Computer Science and Engineering, University of California, Riverside, CA 92521, USA; 5 MOE Key Lab of Bioinformatics and Bioinformatics Division, BNRIST/Department of Computer Science and Technology, Tsinghua University, Beijing 100084, China; 6 Institute of Integrative Genome Biology, University of California, Riverside, CA 92521, USA; 7 MOE Key Laboratory of Bioinformatics, Tsinghua University, Beijing 100084, China

**Keywords:** Pseudouridylation, Convolution neural network, Sequence motif, Translation, RNA stability

## Abstract

Pseudouridine (Ψ) is the most prevalent post-transcriptional RNA modification and is widespread in small cellular RNAs and mRNAs. However, the functions, mechanisms, and precise distribution of Ψs (especially in mRNAs) still remain largely unclear. The landscape of Ψs across the transcriptome has not yet been fully delineated. Here, we present a highly effective model based on a convolutional neural network (CNN), called PseudoUridyLation Site Estimator (PULSE), to analyze large-scale profiling data of Ψ sites and characterize the contextual sequence features of **pseudouridylation**. PULSE, consisting of two alternatively-stacked convolution and pooling layers followed by a fully-connected neural network, can automatically learn the hidden patterns of pseudouridylation from the local sequence information. Extensive validation tests demonstrated that PULSE can outperform other state-of-the-art prediction methods and achieve high prediction accuracy, thus enabling us to further characterize the transcriptome-wide landscape of Ψ sites. We further showed that the prediction results derived from PULSE can provide novel insights into understanding the functional roles of pseudouridylation, such as the regulations of RNA secondary structure, codon usage, **translation**, and **RNA stability**, and the connection to single nucleotide variants. The source code and final model for PULSE are available at https://github.com/mlcb-thu/PULSE.

## Introduction

Pseudouridine (Ψ) is known as the most abundant and earliest discovered modified ribonucleoside among more than 100 types of RNA post-transcriptional modifications that have been identified so far [1], [2], [3]. Because of its prevalence in cellular RNAs, it has also been considered as the fifth ribonucleoside [1]. The properties, chemical structure, and distribution of a Ψ are quite different from those of its parental base (*i.e.*, uridine) [4]. Compared to a uridine, the chemical conformation of a Ψ allows the formation of an extra hydrogen bond at its non-Waston-Crick edge [5]. This fact indicates that a Ψ can form a more stable base stacking conformation [6], which is believed to play an important role in stabilizing RNA structure [7]. In ribosomal RNAs (rRNAs), it has been found that Ψs are required for the maintenance of ribosome–ligand interactions and translational fidelity [8]. In addition, Ψs in transfer RNAs (tRNAs) can reduce the conformational mobility of the structural elements around the modified sites and thus affect the amino acid transfer efficiency [9]. Such a stabilization function of Ψs in anticodon stem-loop of tRNA^Lys,3^ has also been validated by Nuclear Magnetic Resonance (NMR) spectroscopy [10]. Moreover, Ψs in spliceosomal RNAs can be involved in the ribonucleoprotein (RNP) assembling and pre-mRNA splicing processes [11]. The aforementioned findings indicate that most likely Ψs are tightly related to RNA structure stabilization, translation process, and RNA stability. Despite these observations, the underlying mechanisms of pseudouridylation in the aforementioned processes still remain to be further explored.

The conversion from a uridine to a Ψ is catalyzed by Ψ synthases (PUSs) through two distinct processes, including RNA-dependent and RNA-independent operations [12]. The RNA-dependent pseudouridylation process relies on the box H/ACA RNPs, which consist of a small box H/ACA RNA and four core proteins, including centromere-binding factor 5 (Cbf5; also known as dyskerin in mammals), non-histone protein 2 (Nhp2), glycine-arginine-rich protein 1 (Gar1), and nucleolar protein 10 (Nop10), to form a pseudouridylation pocket for substrate recognition and catalytic activity [13]. In the RNA-independent pseudouridylation process, a single synthase protein, such as PUS7, is responsible for both substrate recognition and pseudouridylation catalysis [12]. So far, about 13 types of PUSs in human have been identified, and generally it is difficult to unveil the consensus catalytic laws of pseudouridylation [14]. Moreover, it has been shown that pseudouridylation in RNAs is highly dynamic and inducible [12], which makes it even harder to characterize the properties of pseudouridylation. On the other hand, RNA modification is mostly a sequence pattern recognition process, as it is heavily dependent on the sequence binding preferences of catalytic proteins [15]. From this point of view, it is reasonable to speculate that RNA pseudouridylation is determined by the sequence contexts of the sites being modified.

To characterize the intrinsic properties of Ψs, we need to develop efficient methods to accurately identify Ψ sites at single-base resolution and obtain a transcriptome-wide map of Ψs. Traditional Ψ detection methods are mainly based on the N_3_-CMC labeling and gel electrophoresis experiments [16], which are often laborious and time-consuming. Recently, several high-throughput profiling techniques, including Pseudo-seq [17], Ψ-seq [18], pseudouridine site identification sequencing (PSI-seq) [19], and N_3_-CMC-enriched pseudouridine sequencing (CeU-seq) [20], have been proposed to map RNA Ψ sites to reference transcriptomes. These high-throughput experiments typically combine CMC derivatives with next-generation sequencing or deep sequencing techniques to detect Ψ sites on a transcriptome-wide scale. However, these experiments are generally costly and often require tremendous time and effort in deriving the positions of Ψ sites. In addition, although recent high-throughput sequencing techniques, such as Ψ-seq and CeU-seq, have been able to identify large-scale Ψ sites in mRNAs, they may still miss numerous modification sites due to their intrinsic limitations (*e.g.*, the incompleteness of CMC-labeling or the read mappability issue). Therefore, efficient computational approaches to identify Ψs accurately are especially needed to complement these experimental methods and facilitate the studies of pseudouridylation. The computational prediction of transcriptome-wide Ψ sites and characterization of their sequence contexts may also provide important hints in understanding the functional roles of pseudouridylation in RNA regulation. Although several computational approaches and web servers, such as PPUS [21] and iRNA-PseU [22], have been developed to predict novel Ψ sites, they either can only be applied to predict PUS-specific sites (*i.e.*, can only predict PUS4-specific sites for human) or need to use the handcrafted features derived from the chemical properties of nucleotides.

Recently, deep learning techniques, especially convolutional neural networks (CNNs), have been widely used in genomic data analyses for extracting accurate sequence features [23], [24], [25]. CNNs were first developed for handwriting recognition and face identification [26], and have become one of the most famous and powerful learning models in the fields of computer vision, speech recognition, and natural language processing [27], [28], [29]. Despite its powerful predictive capacity, it remains unknown whether a CNN model can be used to effectively capture the contextual sequence features of pseudouridylation and accurately predict new Ψ sites.

In this study, we have developed a computational framework, called PseudoUridyLation Site Estimator (PULSE), to predict novel Ψ sites from large-scale profiling data of Ψs based on the sequence contexts of target sites. To our knowledge, our study is the first deep learning-based attempt to characterize the contextual sequence features of pseudouridylation by fully exploiting the currently available large-scale profiling data of Ψs. PULSE employs a CNN model, which contains two alternately-stacked convolution and pooling layers responsible for local feature extraction from the input contextual sequences and two fully-connected layers responsible for feature integration and estimation of the pseudouridylation potential of a candidate site. Tests on both human and mouse data have demonstrated that PULSE can achieve high prediction accuracy and significantly outperform other state-of-the-art prediction approaches. The new sequence features captured by PULSE are not only consistent with the recognized motifs of known PUSs, but also match the binding patterns of several nucleotide-binding proteins, which may provide useful hints for discovering new potential PUSs or associating proteins. In addition, the underlying sequence contexts of Ψs detected by PULSE offer an effective indicator to investigate the functional effects of single nucleotide variants (SNVs) on pseudouridylation, which may help reveal possible associations between pseudouridylation and complex diseases. Moreover, the trained PULSE model allows us to unveil the transcriptome-level characteristics of pseudouridylation. The prediction results of PULSE provide several new insights about the functional roles of pseudouridylation. For example, pseudouridylation is codon biased and rare codons are more likely to be pseudouridylated to achieve optimal mRNA translation. Also, our integrative analysis of ribosome profiling data demonstrated that pseudouridylation is involved in modulating the translation initiation and elongation processes. These results indicated that the predictions of PULSE may shed light on the underlying mechanisms and functional roles of pseudouridylation in post-transcriptional regulation.

## Method

### Data collection and pre-processing

The Ψ modification site data were downloaded from the RMBase database [30] which includes the high-throughput profiling data of Ψs collected from three recent experimental studies [17], [18], [20]. All the labeled Ψ sites were separated into a human dataset and a mouse dataset. In addition, the overlap between human and mouse datasets which represents the conserved Ψ sites was considered as a relatively reliable dataset for further model validation. Moreover, the Ψ sites which were identified by SCARLET from the recent experimental study [20] were also used for assessing the prediction accuracy of PULSE. All of the aforementioned modification sites were mapped to the reference genome (human: hg19; mouse: mm10) and those that cannot be mapped to thymines were discarded. A sequence of 101-nt length that covers the Ψ site and has a 50-nt window flanking on its both sides was labeled as a positive sample, while the sequence of the same length that is centered at a thymine that is closest to a corresponding Ψ site and does not have any overlap with any positive sample was labeled as a negative sample. In the end, we collected 7720 and 6166 samples in total for human and mouse, respectively. The ratio of positive and negative samples was close to 1 (human: 3901 positive samples *vs*. 3819 negative samples; mouse: 3057 positive samples *vs*. 3109 negative samples). The sequence samples were then encoded into binary matrices as the input to our model using the one-hot encoding scheme. For the imbalanced testing datasets with 1:*n* positive-to-negative ratio (PNR), the positive samples were collected using the same way as we described above, while the negative samples were collected from the nearest *n* thymine sites that have no overlap with any positive samples.

### Model design

**Figure 1:**
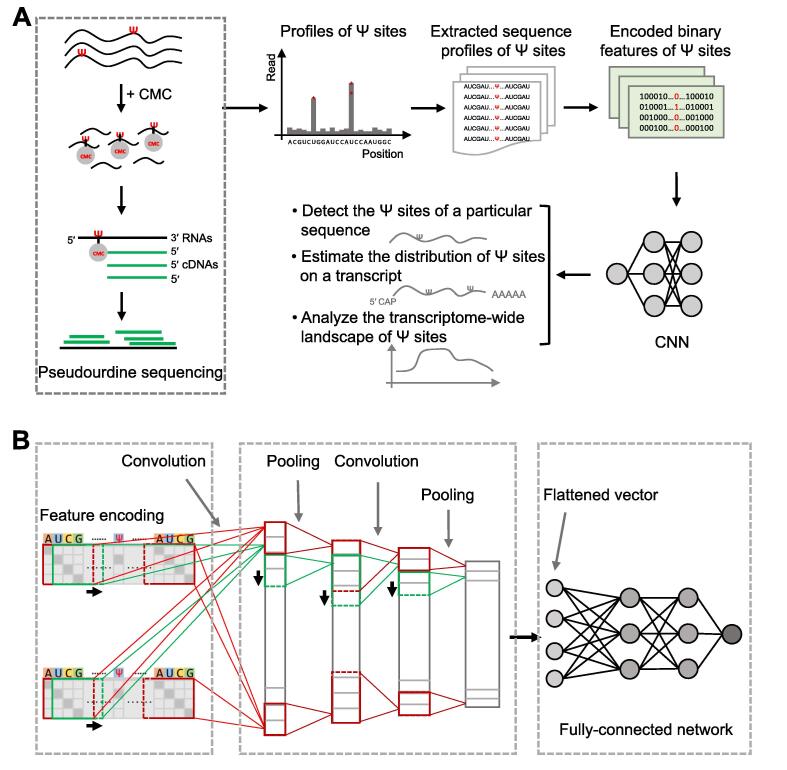
**Overview of the PULSE pipeline** **A.** The schematic flow diagram of PULSE. The Ψ sites can be experimentally profiled by high-throughput sequencing techniques, such as CeU-seq, Pseudo-seq, Ψ-seq, and PSI-seq. PULSE first extracts the sequence profiles of a potential Ψ site (*i.e.*, the region within 50 nt upstream and downstream of the target site) and encodes them into binary features, which are then fed as input data to a particularly designed CNN model. After parameter learning, the trained model is used for downstream analyses, such as detecting the Ψ sites of a given RNA sequence, estimating the distribution of Ψ sites on a transcript, and elucidating the transcriptome-wide landscape of pseudouridylation. **B.** The CNN architecture used in PULSE. Two convolution layers and two pooling layers are first alternately-stacked and used for feature detection, and then a fully-connected network with two hidden layers is added for global feature evaluation and Ψ potential estimation. Given a uridine site of interest and its sequence context, PULSE outputs a Ψ potential score which basically represents the likelihood of pseudouridylation for this target site. PULSE, PseudoUridyLation Site Estimator; Ψ, pseudouridine; CeU-seq, N_3_-CMC-enriched pseudouridine sequencing; PSI-seq, pseudouridine site identification sequencing; CNN, convolutional neural network.

We have designed a computational pipeline to fully characterize pseudouridylation (Figure 1A). To encode the contextual sequence features of a potential Ψ site of interest, we first extend the target site both upstream and downstream by 50 nt and then use a simple four-dimensional binary vector to encode each nucleotide (Figure 1A; File S1). Then, the encoded matrix of an input contextual sequence is fed into a particularly-designed CNN model to capture the latent features of the potential sequence determinants of a Ψ site. Our CNN model consists of two alternately-stacked convolution and pooling layers followed by a two-layer fully-connected network (Figure 1B; File S1). In particular, the convolution kernels from the convolution layers scan the input matrix that encodes the input sequence profiles and capture intrinsic hidden features about the local contextual patterns of the target site. The last fully-connected layer (also called the output layer) employs a softmax function to perform the classification task.

Overall, for a given sequence *l*, the information flow of PULSE can be abstracted into the following equation:

*lPPS(l) = softmax(net_2_(acti_3_(net_1_(pool_2_(acti_2_(conv_2_(pool_1_(acti_1_(conv_1_(l))))))))))*

where *lPPS(l)* represents the final prediction score of the target site, *conv_i_()*, *acti_i_()*, *pool_i_()*, and *net_i_()* stand for the *i*-th convolution, neuron activation, pooling, and full-connection operations, respectively. In our framework, the length of the input sequence *l* is set to 101, as we extend the target site both upstream and downstream by 50 nt. We used grid search with a cross-validation procedure [31] to calibrate the hyperparameters of our CNN model (see the “Training procedure and model evaluation” section).

### Training procedure and model evaluation

The core of PULSE is a CNN consists of two alternately-stacked convolution and pooling layers and two fully-connected layers. During convolution, the input matrices of dimension *L* × 4 (where *L* stands for the length of the input sequences) are first cross-correlated with several convolution filters and then the convolved outputs are rectified by a Parametric Rectified Linear Unit (PReLU) activation function [32]. In the pooling stage, the pooling operators are applied to the previous convolution and activation results for further motif extraction. After that, the pooled results are flattened to a vector which is then fed to a fully-connected neural network for final classification. In the final setting of PULSE, the sizes of the first and the second convolution layers are set to 4 × 8 and 1 × 8, respectively, and the sizes of both pooling layers are set to 1 × 2. The numbers of convolution operators in the first and second layers are set to 64 and 32, respectively, and the numbers of units in the hidden layers of the fully-connected neural network are set to 64-64-1. We apply a 10-fold cross-validation strategy to determine the best values of hyperparameters, including the filter sizes, the filter numbers, the learning rate, the dropout probability, and the number of training epoches, and evaluate the prediction performance of our model. In particular, we first randomly separate entire data into 10 folds. For each fold, the held-out 1/10 dataset is used as testing data and the remaining 9/10 is used as training data. Meanwhile, in each fold, we further divide the training data into 10 subfolds, and run another (nested) 10-fold cross-validation procedure to choose the optimal settings of hyperparameters based on the trained model over 9/10 subsets and the evaluation result on the held-out 1/10 subset of the training data. After the optimal hyperparameters are determined using this nested cross-validation procedure, all the training data are merged and used to train the model and then evaluate the prediction performance on the held-out testing data in each fold. All the hyperparameters except the number of training epoches are computed through grid search. The prediction results over the 10 folds are collected together as the final prediction results. The human and mouse datasets are used independently to train two separate PULSE models (*i.e.*, hPULSE and mPULSE). In our model, the hyperparameters of hPULSE and mPULSE are almost the same except the number of training epoches.

PULSE is implemented based on the Keras library (https://keras.io) in Python. Back propagation is applied in the training process for efficiently updating the parameters. In addition, several optimization techniques, including stochastic gradient descent (SGD), dropout, batch normalization, early stopping, and momentum, are used to improve the training process (*e.g.*, reducing the likelihood of overfitting).

### Motif visualization and analysis

We apply the filters embedded in the first convolution layer of our CNN model to generate the sequence motifs of pseudouridylation, using the same strategy as previously described [23], [33]. More specifically, we use a window of the size equal to the length of the filters (*i.e.*, 8) to scan the flanking regions on both sides of a Ψ site. During this scanning process, those sequence segments (of length 8) with activation values more than half of the maximum score are output. Then these detected sequence segments are converted into the position weight matrix (PWM) form to generate the corresponding motifs representing the sequence contexts of pseudouridylation. To compare these obtained motifs to those known binding patterns of RNA-binding proteins (RBPs) and transcription factors (TFs), we search over the CIS-BP [34] and HOCOMOCO [35] databases (version 2016 for both) using the Tomtom tool [36], respectively, and then cluster all the motifs using RSAT [37] with default parameter settings. The final sequence motifs are visualized using Seq2Logo [38]. We also sort out all the generated motifs and perform a clustering analysis on them (File S1).

### Transcriptome-wide detection of Ψ sites

We further apply PULSE to detect potential Ψ sites on each transcript along the genome. All the RNA sequences of human and mouse were downloaded from Ensembl by Biomart under references hg19 and mm10, respectively. For each transcript, every thymine site and the flanking 50-nt regions on its both sides are extracted as the input sequence profile to PULSE (‘N’s are padded if the flanking windows are out of the transcripts). Then PULSE computes the local pseudouridylation potential score (lPPS) for each thymine, which measures its pseudouridylation probability.

### Transcript pseudouridylation potential score

To evaluate the pseudouridylation potential of a particular transcript, *i.e.*, the estimation of the overall pseudouridylation level of a complete transcript, we defined a new metric called the transcript pseudouridylation potential score (tPPS). In particular, for a transcript *s*, its tPPS value can be defined as follows:

$$\text{tPPS}\left(s\right)=\frac{num\left(\Psi \right)/num\left(U\right)}{K/L}$$

where

$$num\left(\Psi \right)=\sum_{k=1}^{K}I\left(\mathrm{lPP}S_{k}>0.5\right)$$

$$num\left(U\right)=\sum_{k=1}^{K}I\left({\mathrm{lPPS}}_{k}\leq 0.5\right)$$

in which *num*() represents a count function, *I*() represents a binary indicator function, *lPPS_k_* stands for the lPPS of the *k*-th Ψ site in *s*, *K* represents the total number of thymines in *s*, and *L* stands for the length of *s*. In the aforementioned equation, the numerator represents the ratio between Ψs and thymines, which thus measures the relative abundance of possible Ψs in a transcript. However, this value may bias to those uridine-enriched transcripts. In order to eliminate such bias, the ratio in the numerator is further normalized by the abundance of both thymines and Ψs in the transcript.

## Results

### Model validation

**Figure 2:**
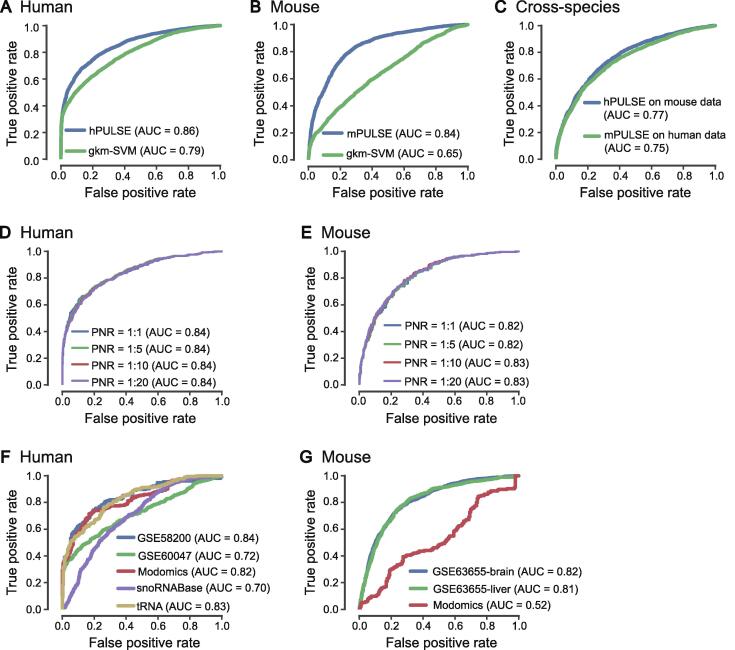
**Performance evaluation of PULSE** **A.** and **B.** The ROC curves and the corresponding AUC scores reported in 10-fold cross-validation for human and mouse, respectively. The terms ‘hPULSE’ and ‘mPULSE’ represent the PULSE models trained based on human and mouse datasets, respectively. gkm-SVM, which is a classical model for sequence classification based on the gapped *k*-mers, was used as a baseline method for comparison. **C.** The results on the cross-species test between human and mouse. Blue and green curves represent the test results on the hPULSE model (which was trained from human data) applied to mouse data and the mPULSE model (which was trained from mouse data) applied to human data, respectively. **D.** and **E.** The test results of hPULSE (D) and mPULSE (E) on the imbalanced datasets with different PNRs (including 1:1, 1:5, 1:10, and 1:20) that did not have any overlap with training data. **F.** and **G.** Performance of the retrained hPULSE (F) and mPULSE (G) on individual held-out datasets. ROC, receiver operating characteristic; AUC, area under the ROC curve; PNR, positive-to-negative ratio.

To evaluate the prediction performance of PULSE, we applied a 10-fold cross-validation procedure on both human and mouse data (see Method). In our training process, the Ψ sites identified from high-throughput profiling experiments and the corresponding flanking regions of 50 nt on both sides of individual Ψ sites were considered positive samples, while uridine sites with flanking windows of 50 nt on both sides that are the closest to some Ψ sites and do not have any overlap with the positive samples were considered as the negative samples. We trained PULSE on human and mouse datasets separately, resulting in two trained models called hPULSE and mPULSE, respectively. We also compared the prediction performance of PULSE to that of a baseline approach, called gkm-SVM, which is a widely-used SVM-based classifier based on gapped *k*-mers that also uses only sequence information [39]. Our 10-fold cross-validation tests showed that both hPULSE and mPULSE can achieve high prediction accuracy, with the area under the receiver operating characteristic curve (AUC) scores 0.86 and 0.84, respectively, which were significantly better than those of gkm-SVM (Figure 2A and B, Figure S1A and B). We further validated PULSE on several small but reliable datasets, which also displayed high prediction accuracy (File S1).

Previous studies showed that the pseudouridylation profiles of transcriptome across human and mouse were conserved to some extent despite the possible difference in the underlying mechanisms of pseudouridylation [20]. Thus, we performed a cross-species test between human and mouse datasets, that is, we used the PULSE model trained from the human (mouse) pseudouridylation profiles to test the mouse (human) data. As expected, such a cross-species test demonstrated a strong conservation relationship between human and mouse in pseudouridylation (Figure 2C, Figure S1C). In addition, this cross-species validation test also implied an impressive generalization capacity of PULSE in predicting new Ψ sites.

So far, our models have shown good performances on balanced datasets (*i.e.*, PNR is 1:1). However, in the real world, usually there are much more unmodified or undetected uridines in RNA transcripts than the modified ones, which means that the previous balanced tests may overestimate the precision of the models. To solve this problem, we retrained our models with the same hyperparameters as we searched before and then tested several imbalanced testing sets with different PNRs (including 1:1, 1:5, 1:10, and 1:20). The tests on these imbalanced datasets also showed competitive performances of our models (Figure 2D and E, Figure S1D and E).

Since the Ψ sites in our training data integrated from several different studies only showed small overlap between each other (Table S1), the trained model may be biased to specific data sources or experiments. To investigate this potential problem and further verify the generalization capacity of our model, we separated all the Ψ sites into independent datasets according to their sources (*i.e.*, GSE58200 [40], GSE60047 [18], GSE63655 [20], snoRNABase [41], and Modomics [2]). Then we held out individual datasets as testing data and retrained our model on the remaining datasets. This additional test on individual held-out datasets also showed a high prediction accuracy of our model except on the Modomics of mouse (Figure 2F and G, Figure S1F and G). The reason that held-out Modomics data as testing data in mouse did not yield good prediction performance was probably due to the lack of Ψ sites in small RNAs in the corresponding training data (Table S2).

We further tested PULSE on three small but relatively more reliable datasets (Tables S3 and S4), which also displayed similar high prediction performance (File S1). In addition, we compared PULSE to other state-of-the-art methods, including PPUS [21] and iRNA-PseU [22]. Specifically, we first directly compared the cross-validation results of our models to those of PPUS and iRNA-PseU evaluated on the whole balanced dataset. The results showed that our models performed much better than the others (File S1). To further compare our models to PPUS and iRNA-PseU on imbalanced datasets, we retrained our models and tested them on four imbalanced datasets (*i.e.*, with PNRs 1:1, 1:5, 1:10, and 1:20, respectively) which did not overlap with the training data. Expectedly, these comparison results also showed that our models performed much better than the others (Table 1). In addition, we further evaluated the performances of PPUS and iRNA-PseU on the aforementioned three reliable datasets, which also supported the superior predictive power of our models over the others (File S1).

**Table 1:** **Comparison between PULSE and the previous methods (PPUS and iRNA-PseU) on independent imbalanced datasets**

**Species**	**Predictor**	**PNR**	**Precision**	**Recall**	**F1-score**	**MCC**
Human	hPULSE	1:1	0.85	**0.65**	**0.736**	**0.547**
		1:5	0.50	**0.65**	**0.566**	**0.471**
		1:10	0.32	**0.65**	**0.432**	**0.382**
		1:20	0.19	**0.65**	**0.289**	**0.291**
	PPUS	1:1	**0.94**	0.09	0.156	0.193
		1:5	**0.92**	0.09	0.156	0.253
		1:10	**0.89**	0.09	0.156	0.261
		1:20	**0.80**	0.09	0.154	0.253
	iRNA-PseU	1:1	0.64	0.59	0.614	0.265
		1:5	0.25	0.59	0.355	0.187
		1:10	0.15	0.59	0.234	0.146
		1:20	0.08	0.59	0.138	0.107
Mouse	mPULSE	1:1	**0.75**	0.74	**0.741**	**0.485**
		1:5	**0.36**	0.74	**0.486**	**0.376**
		1:10	**0.23**	0.74	**0.350**	**0.310**
		1:20	**0.13**	0.74	**0.220**	**0.235**
	iRNA-PseU	1:1	0.65	**0.76**	0.702	0.359
		1:5	0.27	**0.76**	0.397	0.258
		1:10	0.16	**0.76**	0.261	0.205
		1:20	0.09	**0.76**	0.154	0.152

*Note*: PNR, positive-to-negative ratio; MCC, Matthews correlation coefficient. The corresponding highest performances are showed in bold.

In summary, the aforementioned validation tests implied that PULSE can effectively recognize the underlying latent features of the sequence contexts of pseudouridylation and thus yield accurate prediction of Ψ sites.

### The sequence contexts of pseudouridylation captured by PULSE

After PULSE was evaluated, we analyzed and visualized the motifs of the sequence contexts of pseudouridylation captured by the filters employed in the first convolution layer of PULSE, using the same strategy as in the previous studies [23], [33]. In particular, we focused on those high-confident motifs that covered more than 1% (about 50) of the positive samples in the training data. As a consequence, we obtained 300 and 272 sequence patterns identified by PULSE for human and mouse, respectively (see Method). These sequence patterns were then clustered into tens of clusters which may imply different subtypes of pseudouridylation (Figure S2; File S1).

**Figure 3:**
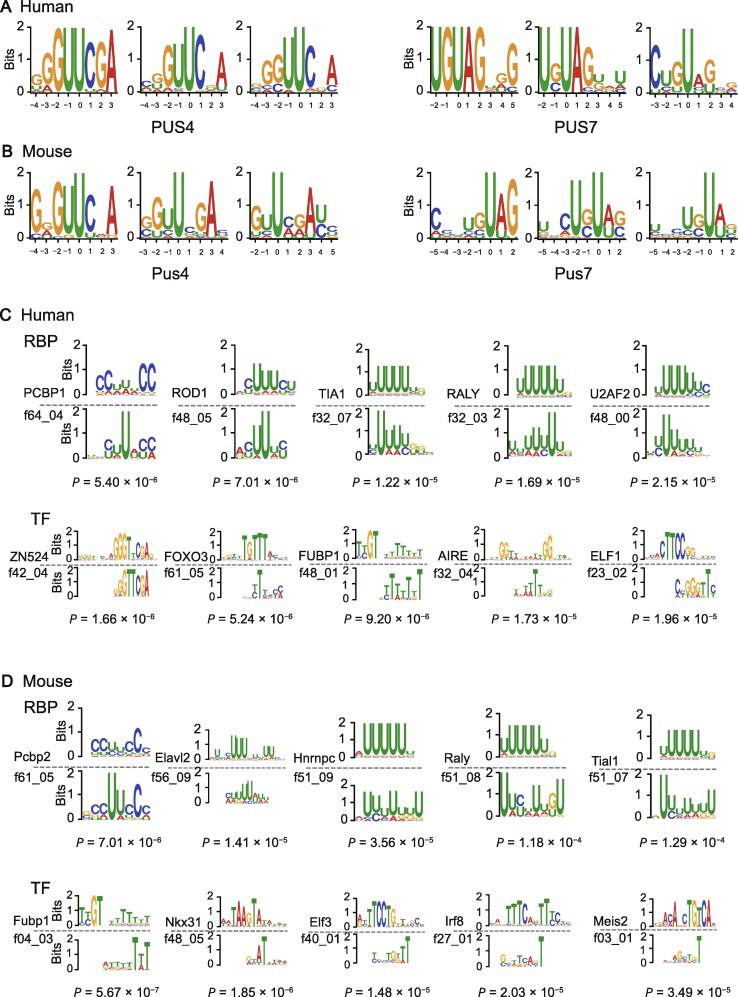
**Examples of the sequence motifs of pseudouridylation identified by PULSE** **A.** and **B.** The sequence motifs of pseudouridylation detected by PULSE corresponding to the known motifs of PUS4 (‘GUΨCNA’) and PUS7 (‘UGΨAG’) for both human (A) and mouse (B). **C.** and **D.** The comparisons between the sequence motifs of pseudouridylation identified by PULSE and the closest matched motifs of RBPs and TFs for human (C) and mouse (D), respectively. Top and bottom show the known binding motifs of RBPs or TFs and the contextual sequence features of pseudouridylation identified by PULSE (the filter IDs are also shown), respectively. PUS, Ψ synthase; RBP, RNA-binding protein; TF, transcription factor.

As expected, we found that the previously known sequence recognition motifs of PUS4 and PUS7 [20], [40], *i.e.*, ‘GUΨCNA’ and ‘UGΨAG’, appeared repetitively in the sequence patterns identified by the filters of our CNN model for both human and mouse (Figure 3A and B). Intriguingly, several novel motifs also appeared repetitively in our visualization results. We hypothesized that these motifs may correspond to other PUSs or recognition proteins. Thus, we mapped our discovered motifs to the known binding motifs of RBPs from the CIS-BP database [34] and TFs from the HOCOMOCO database [35] (see Method). As a result, several of these newly discovered sequence motifs of pseudouridylation significantly matched the known binding motifs of nucleotide-binding proteins for both human and mouse (*P <* 1 × 10^−3^; Figure 3C and D). We found that these matching motifs were highly related to important RBPs and TFs, *e.g.*, PCBP1 (an RBP involved in the regulation of alternative splicing [42]) and FOXO3 (a TF acting as a trigger of apoptosis [43]). Moreover, our model also captured the RNA-binding motif of U2AF, which has been reported to lead to a splicing defect when failing to recognize the pseudouridylated polypyrimidine tract [44]. These discovered novel motifs that matched the known binding sequence patterns of RBPs implied that the corresponding RBPs may play important functional roles in the pseudouridylation process, which thus may also provide new candidate molecules of PUSs for further experimental studies. Since previous studies have shown that RNAs can also be co-transcriptionally modified [45], the TFs with the matching sequence motifs may be related to the triggers of pseudouridylation during RNA transcription.

### The transcriptome landscape of Ψs characterized by PULSE

Each uridine in a transcript can be characterized by an lPPS derived from the trained PULSE model based on its corresponding sequence context. Basically, this lPPS value measures the probability that a uridine can be converted into a Ψ. Based on the distribution of uridines on a transcript and the corresponding lPPS profiles, we derived a new metric, called tPPS (see Method), to estimate the overall pseudouridylation level of this transcript. Based on the lPPS and tPPS profiles derived from PULSE, we are able to study the nucleotide- and transcript-level landscapes of pseudouridylation, respectively.

**Figure 4:**
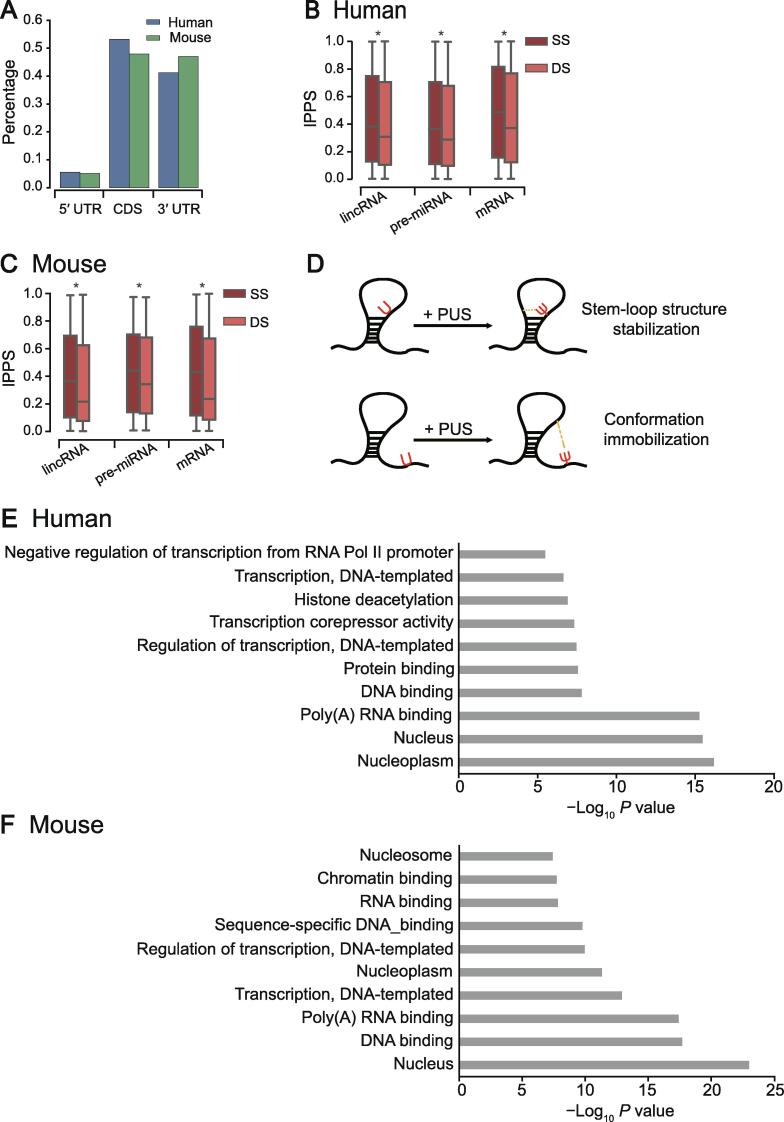
**The transcriptome-level characteristics of pseudouridylation predicted by PULSE** **A.** Distributions of the Ψ sites identified by PULSE over different types of regions in mRNAs. **B.** and **C.** Comparisons of the lPPS values between SS and DS regions over different types of RNAs (including lincRNAs, pre-miRNAs, and mRNAs) for human (B) and mouse (C), respectively. *, *P* < 1 × 10^−200^, rank-sum test. **D.** Schematic illustration of the potential functional roles of Ψ sites in stabilizing RNA secondary structures. **E.** and **F.** GO enrichment analyses of genes with high tPPS values (top 500) carried out by DAVID for human (E) and mouse (F), respectively. lPPS, local pseudouridylation potential score; SS, single-strand; DS, double-strand; GO, Gene Ontology; tPPS, transcript pseudouridylation potential score.

To examine the transcriptome-wide distribution of pseudouridylation (see Method for transcriptome-wide detection of Ψ sites) across different genomic regions, we compared the percentages of pseudouridylation predicted by PULSE among different types of regions, including 5′ UTRs, CDS regions, and 3′ UTRs. Our comparison showed that Ψs appear primarily in the CDS regions (∼ 50%) and the 3′ UTRs (∼ 40%) of both human and mouse mRNAs (Figure 4A), which was consistent with the previous reports [20], [40]. Considering that the region length and the uridine content may affect the distributions of Ψs in different mRNA regions, we also normalized the aforementioned proportions by the region length and corresponding Ψ ratio (Ψ/T), and observed the similar trend (Figure S3). As the 3′ UTRs of mRNAs are tightly associated with RNA stability and translational control [46], [47], it is reasonable to hypothesize that the pseudouridylation activities in the 3′ UTRs are involved in RNA stability modulation and translational regulation.

Pseudouridylation in RNAs has been considered to play important roles in secondary structure stabilization [4], [6]. The structural functions of Ψs in rRNAs and tRNAs have already been relatively well studied [10], [48], [49], [50], and a noticeable observation in the previous studies was that the experimentally detected Ψs are largely located in the loop regions of an RNA secondary structure. However, the functional roles of Ψs in the structures of other types of RNAs remain poorly understood. Here, we are interested in whether Ψs in other types of RNAs play the same roles in the regulation of RNA secondary structures. We first used the RNAfold software [51] to predict the RNA secondary structures of individual sequences centered at putative Ψ sites of the transcripts with high tPPS values (top 500) predicted by PULSE (see Method). We then compared lPPS profiles between single-strand (SS) and double-strand (DS) regions over different types of RNAs, including lincRNAs, pre-miRNAs, and mRNAs, whose structures are generally more diverse than those of tRNAs and rRNAs. We found that Ψs prefer to occur in SS regions over DS regions in all three types of RNAs for both human and mouse [Figure 4B and C; *P* < 1 × 10^−200^, rank-sum test; *n*_(human,lincRNA,SS)_ = 20,918, *n*_(human,lincRNA,DS)_ = 37,852, *n*_(human,pre-miRNA,SS)_ = 3302, *n*_(human,pre-miRNA,DS)_ = 7597, *n*_(human,mRNA,SS)_ = 16,717, *n*_(human,mRNA,DS)_ = 34,357, *n*_(mouse,lincRNA,SS)_ = 28,260, *n*_(mouse,lincRNA,DS)_ = 48,402, *n*_(mouse,pre-miRNA,SS)_ = 3355, *n*_(mouse,pre-miRNA,DS)_ = 7194, *n*_(mouse,mRNA,SS)_ = 18,193, *n*_(mouse,mRNA,DS)_ = 32,270]. In addition, we applied our model to predict the profiles of tRNAs annotated by tRNAscan-SE [52], and found that Ψs also prefer to occur in SS regions of human tRNAs [Figure S4; *P* = 4.30 × 10^−31^, rank-sum test; *n*_(human,SS)_ = 6357, *n*_(human,DS)_ = 4513]. For mouse tRNAs, we did not observe the same trend [Figure S4; *n*_(mouse,SS)_ = 4704, *n*_(mouse,DS)_ = 3555], which was probably due to the lack of Ψ sites on small RNAs in the mouse training data (Table S2). We also looked into the predicted Ψ sites of a tRNA corresponding to alanine (tRNAdb ID: tdbR00000017) and compared them to those experimentally validated sites. We found that our model exactly detected two experimentally reported Ψ sites and one potential novel site (Figure S5). Overall, our analysis results were mostly in line with the previous known distributions of Ψs in tRNAs [8], [10]. Such similar patterns of Ψ distributions in RNA secondary structures implied that most likely the functional roles of pseudouridylation in regulating RNA structures are generic across all types of RNAs.

To explain the discrepancy in the distributions of Ψs in different types of RNA secondary structures, we hypothesize that Ψs may play an important role in stabilizing or rigidifying RNA secondary structures (Figure 4D). Such a hypothesis is also supported by the previous studies [4], [6], [7], [10] which have shown that Ψs in SS RNAs may interact relatively more easily with other nucleotides to constrain the corresponding loop regions and form more stable conformations. More specially, Ψs in an inner loop (*e.g.*, hairpin loop or internal loop) may pair with nearby nucleotides in space to help stabilize the loop structure, while Ψs in external or flanking SS regions may contribute to supporting and immobilizing their proximal inner loops. Although we cannot rule out that this phenomenon may be caused by the bias of the antibodies used in Ψ identification experiments, which may have different binding affinities to the Ψ sites between RNA SS and DS regions, our new analysis results covered pre-miRNAs, lincRNAs, and mRNAs, and previous known distributions in tRNAs and rRNAs strongly supported our hypothesis.

Moreover, the Gene Ontology (GO) enrichment analyses for the top 500 genes with the highest tPPS values for both human and mouse showed that genes with high tPPS values predicted by PULSE are mainly distributed in the nucleus and contribute to DNA or RNA binding (Figure 4E and F; File S1). This phenomenon implied that Ψs in mRNAs may also enhance the bindings between nucleotide-binding proteins and RNAs by increasing their interaction strength and forming more stable complex conformations, which was consistent with the previous results about the potential functions of pseudouridylation in RNA secondary structure and translational regulation [4], [6], [53].

### Pseudouridylation serves as an additional factor in controlling mRNA stability

**Figure 5:**
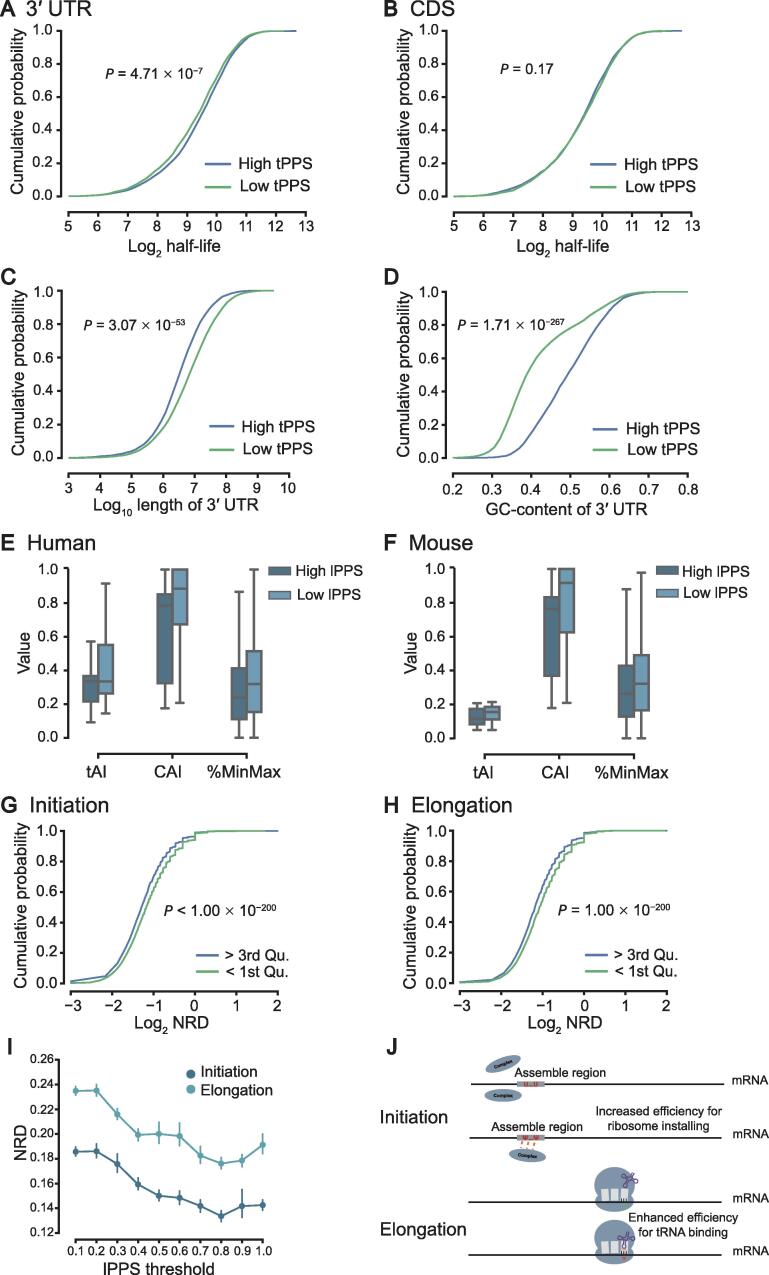
**The functional roles of pseudouridylation inferred by PULSE** **A.** Comparison of the cumulative distribution curves of log_2_ half-life values between two groups of mRNAs with the tPPS values of the 3′ UTRs greater and less than the average level, respectively. *P* = 4.71 × 10^−7^, rank-sum test. **B.** Comparison of the cumulative distribution curves of log_2_ half-life values between two groups of mRNAs with the tPPS values of the CDS regions greater and less than the average level, respectively. *P* = 0.17, rank-sum test. **C.** Comparison of the cumulative distribution curves of log_10_ length of 3′ UTR values between two groups of mRNAs with the tPPS values of the 3′ UTRs greater and less than the average level, respectively. *P* = 3.07 × 10^−53^, rank-sum test. **D.** Comparison of the cumulative distribution curves of GC-contents of 3′ UTR between two groups of mRNAs with the tPPS values of the 3′ UTRs greater and less than the average level, respectively. *P* = 1.71 × 10^−267^, rank-sum test. **E.** and **F.** Comparisons of the tAI, the CAI, and the codon rareness (measured by the %MinMax score) between codons from groups with lPPS values greater than the average level (termed by ‘High lPPS’) and with lPPS values less than the average level (termed by ‘Low lPPS’) for human (E) and mouse (F), respectively. *, *P* < 1 × 10^−200^, rank-sum test. **G.** and **H.** Comparisons of the cumulative distribution curves of log_2_ NRD values between positions with greater than 75% quantile (termed by ‘> 3rd Qu.’) and lower than 25% quantile (termed by ‘< 1st Qu.’) of lPPS values for translation initiation (G) and translation elongation (H) in human, respectively. **I.** NRDs of translation initiation and elongation at different lPPS thresholds. **J.** The putative models proposed to explain the functional roles of pseudouridylation in translation initiation and translation elongation, respectively. In translation initiation, Ψs in ribosome assemble regions may help the translation initiation complexes bind to mRNAs mainly due to the extra hydrogen donors resulting from pseudouridylation. In translation elongation, Ψs located in the A sites of ribosomes may enhance the loading of tRNAs, which may thus promote the movement of ribosomes. tAI, tRNA adaptation index; CAI, codon adaptation index; NRD, normalized ribosome density.

Previous studies have suggested that pseudouridylation may play an important role in enhancing mRNA stability [4], [18], which is probably modulated through the 3′ UTRs of mRNAs. To examine more details of this issue, we analyzed the potential relationships between the predicted pseudouridylation potentials of the 3′ UTRs of mRNA transcripts and their half-lives. In particular, we first applied PULSE to compute the tPPS values of the 3′ UTRs for those transcripts with known half-life information (File S1). We then divided these transcripts into two groups, with the tPPS values of 3′ UTRs greater or less than the average level, respectively. The comparison between these two groups showed that mRNAs with higher tPPS values of 3′ UTRs tend to have relatively longer half-lives [Figure 5A; *P* = 4.71 × 10^−7^, rank-sum test; *n*_(High tPPS)_ = 3215, *n*_(Low tPPS)_ = 4260]. We also performed a similar analysis on the relationships between the tPPS values of CDS regions and mRNA half-lives, but did not observe any significant effect of Ψs in the CDS regions on mRNA half-lives [Figure 5B; *P* = 0.17, rank-sum test; *n*_(High tPPS)_ = 3264, *n*_(Low tPPS)_ = 4217]. These results indicated that Ψs in the 3′ UTRs of mRNAs may improve their stability, which was also supported by the previous study [53]. On the other hand, previous studies also reported that the length and GC-content of the 3′ UTR of an mRNA can affect its stability, that is, short 3′ UTR and high GC-content can promote mRNA decay through distinct mechanisms [54], [55]. To decouple the effects of these two factors and pseudouridylation on RNA stability, we also investigated the relationships between the tPPS values of 3ʹ UTRs and their lengths and GC-contents. As a result, we observed that those 3′ UTRs with larger tPPS values are significantly shorter and tend to have higher GC-contents [Figure 5C and D; *P* = 3.07 × 10^−53^ for the length of 3ʹ UTR, *P* = 1.71 × 10^−267^ for the GC-content of 3ʹ UTR, rank-sum test; *n*_(High tPPS)_ = 3215, *n*_(Low tPPS)_ = 4260], which implied that Ψs in 3′ UTRs can compensate for the down-regulation effects of RNA stability caused by their short lengths and high GC-contents. To further verify this relationship, we performed additional analyses on several other curated RNA half-life datasets. The additional analysis results suggested that the relationship mentioned above is probably robust to different cell lines (Figure S6; File S1).

### Pseudouridylation fine-tunes the effects of codon bias to maintain translation efficiency

It has been reported that Ψs in mRNAs may affect their translation fidelity [12]. Moreover, when uridines in stop codons are pseudouridylated, ribosomes may read through without translation termination, that is, tRNAs can also bind to these modified stop codons and continue the translation process [56]. These previous studies highlighted the potential regulatory functions of Ψs in translation through changing the properties of the corresponding codons. The distinct distributions of lPPS values of uridines in different codons at individual positions may also support the aforementioned potential regulatory functions of pseudouridylation (Figures S7–S9). Here, we investigated the relationships between the lPPS values of individual uridine-containing codons and different indices of codon usage bias, including the tRNA adaptation index (tAI) [57], the codon adaptation index (CAI) [58], and the %MinMax metric (Table S5; File S1). In general, RNA regions with loose structures where ribosomes can move forward relatively more rapidly tend to have higher tAI and CAI values. On the other hand, pseudouridylation may act as a stumbling block to impede ribosome movement by increasing the rigidity of local conformations during a translation process. Thus, we speculate that pseudouridylation is more likely to happen in codons with relatively lower tAI and CAI values. To validate this speculation, we divided all codons into two groups according to their lPPS values, *i.e.*, based on whether their lPPS values were greater than the average level or not. We then compared the tAI and CAI values of the codons between these two groups, respectively. As a result, codons with higher lPPS values displayed significantly lower tAI and CAI values for both human and mouse (Figure 5E and F; *P* < 1 × 10^−200^, rank-sum test). These results implied that pseudouridylation prefers to occur in relatively rare codons or codons with lower supply of their corresponding tRNAs. Furthermore, for codon rareness, we considered the %MinMax metric that has been developed to evaluate the relative rareness of codons in a coding sequence [59]. We performed the same analysis for %MinMax as we conducted for tAI and CAI. The comparison of the %MinMax values between codons with high and low lPPS values demonstrated that Ψs also prefer to occur in relatively rare codons (Figure 5E and F; *P* < 1 × 10^−200^, rank-sum test), which was consistent with our previous results. In summary, our analysis results showed that pseudouridylation prefers to occur in rare codons, which suggested that pseudouridylation may be involved in controlling the rhythm of translation and perhaps the co-translational folding of the nascent peptide chains.

### Pseudouridylation modulates the translation initiation and elongation processes

It has been revealed that pseudouridylation in mRNAs can enhance the translational capacity [53], which implys that Ψs may play an important role in mRNA translation. Previously, we showed that pseudouridylation is codon biased and may be involved in translation regulation through codon fine-tuning. To further investigate whether pseudouridylation may participate in the modulation of translation rates, we also performed an integrative analysis by combining PULSE prediction results with ribosome profiling data that describe the translation initiation and elongation processes (File S1).

To reveal the functional roles of pseudouridylation in translation initiation, we first collected the human ribosome profiles of translation initiation positions (File S1) and selected initiation codon sites near uridines (*i.e.*, within the range of +/−1 codon). After that, we ran the PULSE model to calculate the lPPS values of the flanking sequences centered at the uridines that were closest to the selected initiation codon sites. Next, we extracted two groups of codons from these selected initiation positions, which had lower than 25% quantile (termed by ‘< 1st Qu.’) and greater than 75% quantile (termed by ‘> 3rd Qu.’) of lPPS values, respectively (File S1). We then compared the normalized ribosome densities (NRDs) of the codon sites in these two groups. The comparison showed that the Ψ sites with higher lPPS values are more likely to be located in regions with lower initiation ribosome densities (Figure 5G; *P* < 1 × 10^−200^, rank-sum test). This result suggested that Ψs in the translation initiation regions may help reduce the accumulation of ribosomes. We speculated that this may be realized by attracting the rRNAs in the small subunits (SSUs) of ribosomes through the formation of extra hydrogen bonds, which can thus accelerate the installing process of initiating ribosomes (Figure 5J).

To decipher the impact of pseudouridylation on the translation elongation process, we explored the human ribosome profiles of translation elongation obtained from a previous study [60], and integrated them with the PULSE prediction results. We first processed the ribosome profiling data using the same strategy as we conducted in the analysis of translation initiation, except that here we mainly focused on the codons in CDS regions. Unsurprisingly, we found that pseudouridylation prefers to occur in the regions with relatively slow elongation rates (Figure 5H; *P* < 1 × 10^−200^, rank-sum test). This result implied that Ψs in mRNAs may help modulate the translation elongation rates. We speculated that Ψs in mRNAs may serve to affect the elongation process by dragging the aminoacyl tRNAs to the A sites of ribosomes during elongation through the force of extra hydrogen bonds (Figure 5J).

To investigate more details about the relationships between pseudouridylation and translation, we further compared the ribosome densities at different lPPS thresholds ranging from 0.1 to 1.0 with an increment of 0.1. We found that the ribosome densities significantly decreased along the lPPS values from 0.1 to 1.0 (Figure 5I). This result demonstrated the robustness of the relationships between pseudouridylation and translation that we previously claimed.

In summary, Ψs in mRNAs are involved in modulating the translation process, including both initiation and elongation, probably by strengthening the bindings of ribosomes or tRNAs to mRNAs. Of course, unveiling the detailed underlying mechanisms will still require further extensive experimental studies.

### Relationships between pseudouridylation and SNVs

**Figure 6:**
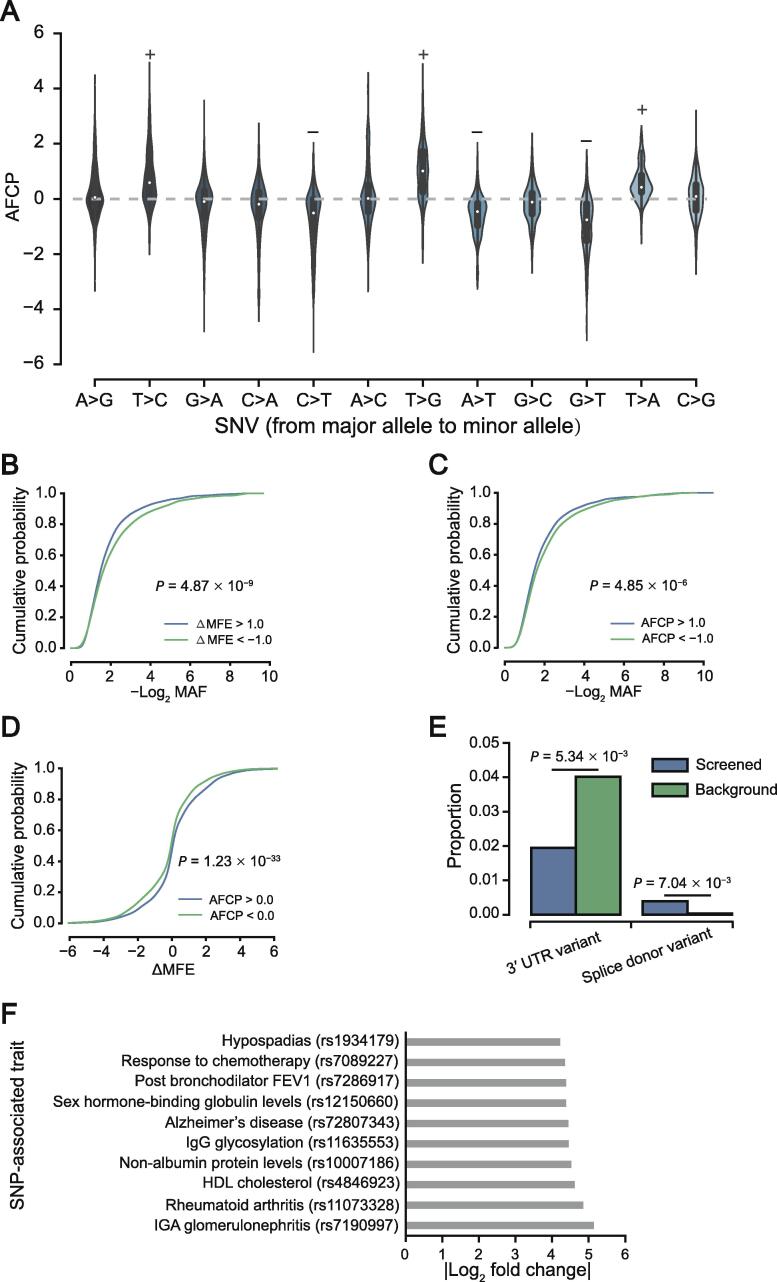
**Relationships between pseudouridylation and SNVs** **A.** Distributions of the AFCP values for different allele pairs. When the T allele was replaced by other nucleotide alleles, the AFCP values were larger than zero (labeled by ‘+’), while when other nucleotide alleles were replaced by the T allele, the AFCP values were smaller than zero (labeled by ‘−’). **B.** Comparison of −log_2_ MAF values between sequences from two groups of SNVs with ΔMFE > 1.0 and ΔMFE < −1.0, respectively. **C.** Comparison of −log_2_ MAF values between sequences from two groups of SNVs with AFCP > 1.0 and AFCP < −1.0, respectively. **D.** Comparison of ΔMFE values between sequences from two groups of SNVs with AFCP > 0.0 and AFCP < 0.0, respectively. **E.** Two types of variants significantly enriched in the screened SNVs with high AFCP values (*i.e.*, |AFCP| > 2). The set of all SNVs used in the analysis was used as the background. **F.** Traits or diseases associated with the SNVs with top 10 highest |AFCP| values. In (B–D), the *P* values were computed by rank-sum test, while in (E), the *P* values were computed by hypergeometric test. SNV, single nucleotide variant; AFCP, allele fold change of pseudouridylation potential; MAF, minor allele frequency; MFE; minimum free energy.

The sequence contexts of Ψs captured by PULSE can enable us to investigate the functional effects of SNVs on pseudouridylation. To demonstrate this point, we first applied PULSE to predict the lPPS profiles of the major alleles and the corresponding minor alleles for SNVs that have been annotated by the current genome-wide association studies (GWAS) and validated by 1000Genomes (File S1). Next, we calculated the log_2_ fold change of lPPS values between major and minor alleles, which was termed as the allele fold change of pseudouridylation potential (AFCP; File S1). Interestingly, we found that when the T allele was replaced by another allele (*i.e.*, A, C, or G), the corresponding lPPS values dropped significantly. On the other hand, when the other alleles were replaced by the T allele, the corresponding lPPS values increased significantly (Figure 6A). This result implied that, a uridine site with a T allele in its contextual sequence is more likely to be pseudouridylated than another one with other alleles in its contextual sequence.

Previous studies showed that SNVs in RNAs may affect stabilities of their secondary structures [61], [62], [63]. To investigate the relationships between pseudouridylation and the effects of SNVs on RNA structure stability, we first used RNAfold [51] to estimate the minimum free energy (MFE) values of the sequences of both major and minor alleles and calculated the change of MFE (denoted by ΔMFE; File S1). We then compared the minor allele frequency (MAF) values between two groups of SNVs, which had ΔMFE greater than 1.0 kCal/Mol and less than −1.0 kCal/Mol, respectively. These two thresholds were chosen according to the 80% and 20% quantiles of all the ΔMFE values. The comparison result showed that alleles with high MAF values are more likely to occur in relatively unstable RNA regions, *i.e.*, with relatively high MFE, which generally correspond to flexible RNA regions, *e.g.*, SS regions [Figure 6B; *P* = 4.87 × 10^−9^, rank-sum test; *n*_(ΔMFE > 1.0)_ = 3248, *n*_(ΔMFE < −1.0)_ = 3347]. From this observation, we speculate that SNVs may act on RNA structures through affecting the pseudouridylation profiles of their corresponding sequences. To study this issue, we first compared the MAF values between two groups of SNVs, which had AFCP values greater than 1.0 and less than −1.0, respectively. This comparison showed that SNVs with larger MAF values are more likely to be pseudouridylated [Figure 6C; *P* = 4.85 × 10^−6^, rank-sum test; *n*_(AFCP > 1.0)_ = 2207, *n*_(AFCP < −1.0)_ = 2568]. In addition, we compared the ΔMFE values between two groups of SNVs with AFCP values greater than 0.0 and less than 0.0, respectively. As a result, we found that alleles with larger predicted pseudouridylation potentials are associated with relatively higher MFE values [Figure 6D; *P* = 1.23 × 10^−33^, rank-sum test; *n*_(AFCP > 0.0)_ = 7853, *n*_(AFCP < 0.0)_ = 8661]. Note that here, although the energy gap (ΔMFE) between major and minor alleles was small (*i.e.*, within a range of ∼ 3 kCal/Mol), it can lead to a dramatic transformation of RNA secondary structure (Figure S10). In addition to the aforementioned results, we also performed similar analyses based on the prediction results from remuRNA [64] and RNAsnp [65], which predict an ensemble of RNA secondary structures, and observed similar trends (Figures S11 and S12). Overall, our analyses indicated that SNVs can affect the pseudouridylation potentials of RNA sequences to change the stability of their secondary structures.

We also performed an enrichment analysis of the variants with relatively high AFCP values (*i.e.*, |AFCP| > 2.0) over different types of genomic regions. The enrichment analysis results showed that SNVs with high AFCP values are significantly enriched in the splice donor regions (Figure 6E; *P* = 7.04 × 10^−3^, hypergeometric test) and depleted in the 3′ UTRs (Figure 6E; *P* = 5.34 × 10^−3^, hypergeometric test), when compared to the background (*i.e.*, the set of all SNVs used in the analysis). These results implied that pseudouridylation may be relatively more sensitive to SNVs in the contextual sequences that are related to RNA splicing and regulatory functions of 3′ UTRs. We also combined the traits associated to the SNVs with the predicted lPPS values to illustrate latent relations between pseudouridylation and important phenotypes, such as complex diseases. In particular, we first selected the top 10 variants with the highest absolute AFCP values, including rs7190997, rs11073328, rs4846923, rs10007186, rs11635553, rs72807343, rs12150660, rs7286917, rs7089227, and rs1934179. We then investigated the disease traits associated with these 10 selected SNVs. Interestingly, we found that these 10 variants were mainly associated with immune system lesions (Figure 6F). For example, rs11073328 and rs11635553 have been reported to be related to rheumatoid arthritis [66] and IgG glycosylation [67], respectively. This result implied that pseudouridylation may play an important role in the immune system.

## Discussion

Based on PULSE we showed that pseudouridylation is codon biased and closely related to RNA translation. We believed that these relationships were constructed during a long evolution process of epigenome. Actually, it has been widely believed that rare codons appear mainly due to the codon usage bias resulting from mutation and natural translation selection [68]. Codon usage plays an essential role in regulating multiple levels of cellular processes, such as translation and protein folding. During the translation process, codons are carefully selected to achieve accurate translation and thus optimal cellular fitness to a certain context, *e.g.*, expression of a certain gene in a certain organism or under certain conditions [69]. Despite that rare codons in an mRNA transcript may decrease its translation efficiency, they are generally important for regulating protein folding and RNA stability [70], [71]. Although codon usage under selection pressure during the evolution process can fine-tune gene expression, it may not be able to have a quick response to a sudden change caused by environmental stimulation. On the other hand, pseudouridylation can provide an additional factor to further fine-tune the translation process. Under a certain cellular condition, pseudouridylation can increase the translation speed of original rare codons probably through strengthening the binding of ribosomes or tRNAs to mRNAs, to ensure the efficient translation of functionally important residues in proteins, when responding to dramatic environmental changes. Those rare codons without such a fine-tuning function may die out during molecular evolution and their functions in the control of translation may be lost. Thus, from an evolutionary point of view, selecting rare codons for pseudouridylation may promote the conservation of certain rare codons in the genome.

Our analyses indicated that pseudouridylation can fine-tune RNA stability. Note that there are many potential biological factors that can affect RNA stability, such as the regulation of RBPs, RNA modification, polyadenylation, and miRNA-mediated regulation. Thus, most likely the half-life of an RNA is influenced by a mixed effect of all these factors. Our results indicated that although with a small effect (Figure 4A), pseudouridylation can significantly contribute to the regulation of RNA stability. Such a finding has an important biological implication. In fact, in the literature, similar phenomena have also been observed. For example, it has been found that *N*^6^-methyladenosine can significantly modulate mRNA translation efficiency, although only with a marginal effect [72].

RNA pseudouridylation is obviously crucial to RNA regulation simply by its prevalence in transcriptome and its high conservation across different species. Therefore, a comprehensive understanding of RNA pseudouridylation will be conducive to the consummate studies of RNA modifications and RNA epigenetics. The studies of RNA pseudouridylation especially in mRNAs may help understand its functional roles in post-transcriptional regulation. Given the complication of the underlying pseudouridylation mechanisms (*e.g.*, there are at least 13 types of PUSs) and the limitations of current experimental profiling techniques, it is generally difficult to explore the biological functions of pseudouridylation through conventional experimental methods (*e.g.*, gene knockdown experiment). This challenge can also partially explain why pseudouridylation is relatively less studied compared to other RNA modifications. Here, our proposed model provides a natural method to unify all current available Ψ profiling data and fully exploit the underlying contextual sequence features of pseudouridylation. Such a strategy can take advantage of the learning and predictive power of the CNN model to reveal the characteristics and potential functional roles of pseudouridylation by connecting the prediction results to the profiles of other biological factors or processes (*e.g.*, RNA secondary structure, translation initiation and elongation), which can provide useful hints into understanding the underlying mechanisms of pseudouridylation.

## Conclusion

In this study, we developed an effective CNN model to detect Ψ sites, based on which we further analyzed the landscape of Ψs across the human and mouse transcriptomes. Our model can not only capture the known motifs of pseudouridylation that were consistent with previous studies, but also reveal novel sequence patterns that may help uncover potential new PUSs. The analysis of the associations between SNVs and the changes of pseudouridylation potentials based on the sequence contexts captured by our model showed that pseudouridylation may be involved in several complex diseases, such as rheumatoid arthritis (associate trait of the rs11073328 SNV) and Alzheimer’s disease (associate trait of the rs72807343 SNV) [73]. Our extensive analysis on the relationships between predicted pseudouridylation scores and different types of RNA secondary structures showed that Ψs are more likely to occur in SS RNA regions rather than DS RNA regions, which led to a speculation that Ψs may act as an anchor to stabilize or rigidify RNA structures. Comparison of half-lives between mRNA transcripts with high and low tPPS values of their 3′ UTRs derived from the prediction results showed that Ψs in the 3′ UTRs of mRNA transcripts may enhance their stability. Also, the GO enrichment analysis of genes with high pseudouridylation scores predicted by our model may provide useful hints for understanding the biological functions of pseudouridylation. We also showed that pseudouridylation is codon biased and uridines in rare codons are more likely to be pseudouridylated, which may serve as an important regulatory strategy for achieving optimal mRNA translation. Comparisons of ribosome occupancy densities between positions with high and low pseudouridylation potentials predicted by our model for both translation initiation and elongation showed that pseudouridylation often occurs in the ribosome sparse regions, which implied that Ψs may promote the translation process by enhancing the interactions between ribosomes and RNAs. We believe that these results can provide novel insights into the studies of pseudouridylation and our computational framework can also inspire studies on other types of RNA modifications.

## Code availability

The source code, the data files used in the analyses, and the final PULSE model can be downloaded from https://github.com/mlcb-thu/PULSE.

## CRediT author statement

**Xuan He:** Conceptualization, Methodology, Software, Formal analysis, Investigation, Data curation, Writing - original draft, Visualization. **Sai Zhang:** Methodology, Supervision. **Yanqing Zhang:** Data curation. **Zhixin Lei:** Validation. **Tao Jiang:** Supervision, Writing - review & editing, Funding acquisition. **Jianyang Zeng:** Conceptualization, Methodology, Writing - review & editing, Supervision, Project administration, Funding acquisition. All authors have read and approved the final manuscript.

## Competing interests

The authors have declared no competing interests.

## Supplementary material

Supplementary File S1 **Supplementary descriptions of methods and results (in PDF format)**.

Supplementary Figure S1 **The precision-recall (PR) curves and the corresponding area under the precision-recall curve (AUPR) scores in the cross-validation results**. **A**. and **B**. Comparisons of the 10-fold cross-validation results between PULSE and the baseline approach gkm-SVM for human and mouse, respectively. **C**. The results on cross-species tests between human and mouse. **D**. and **E**. The precision-recall curves and the corresponding AUPR scores of the models on the independent imbalanced datasets with different positive-to-negative ratios (PNRs; including 1:1, 1:5, 1:10, and 1:20) that did not have any overlap with training data. **F**. and **G**. The precision-recall curves and the corresponding AUPR scores of the retrained models on individual held-out datasets for human and mouse, respectively. The legends are the same as in Figure 2.

Supplementary Figure S2 **Sequence motif clustering**. **A**. and **B**. The clustering heatmaps of the sequence motifs of pseudouridylation identified by PULSE for human (A) and mouse (B). The sequence motifs of human and mouse had 70 and 69 clusters, respectively. The clusters are labeled by different colors.

Supplementary Figure S3 **The normalized distributions of the predicted Ψ sites in different genomic regions**. **A**. and **B**. The proportions of the predicted Ψ sites were normalized by the lengths of individual regions and their Ψ/T ratios, respectively.

Supplementary Figure S4 **Biased distributed pseudouridines in tRNA structures**. Comparisons of the lPPS scores computed by PULSE in tRNAs between single-strand (SS) and double-strand (DS) regions. *, 10^-10^ < *P* < 10^-5^; ***, *P* < 10^-30^, rank-sum test.

Supplementary Figure S5 **Known and novel pseudouridylation sites detected in a tRNA**. PULSE accurately detected two experimentally reported pseudouridylation sites (marked by red dots) and one novel site from all 15 uridine candidates (all the three detected sites had lPPS scores above 0.5) in a tRNA (tRNAdb ID: tdbR00000017) corresponding to alanine.

Supplementary Figure S6 **Relationships between RNA half-lives and pseudouridylation**. Relationships between RNA half-lives and pseudouridylation of seven human lymphoblastoid cell lines (GM07019, GM07029, GM10835, GM12812, GM12813, GM12814, and GM12815) (**A–G**) and the mouse Neuro-2a cell line (**H**).

Supplementary Figure S7 **Pseudouridine potentials of all uridine-containing condons**. The lPPS values predicted by PULSE for all uridine-containing codons for both human (**A**) and mouse (**B**). The average lPPS value (termed by ‘Avg’) is also shown.

Supplementary Figure S8 **Pseudouridine potentials of human uridine-containing codons with uridine in different nucleotide positions**. The lPPS values predicted by PULSE for the uridine-containing codons with pseudouridylation in different nucleotide positions for human. The average lPPS value (termed by ‘Avg’) is also shown.

Supplementary Figure S9 **Pseudouridine potentials of mouse uridine-containing codons with uridine in different nucleotide positions**. The lPPS values predicted by PULSE for the uridine-containing codons with pseudouridylation in different nucleotide positions for mouse. The average lPPS value (termed by ‘Avg’) is also shown.

Supplementary Figure S10 **The small change of free energy in a range of +/- 3 kCal/Mol can lead to a dramatic transformation of RNA secondary structure**. **A**.–**B**. Cumulative curves of the p-values of the max base pairing distance (*d*_max_) and the minimum coefficient (*r*_min_) calculated by RNAsnp between the allele pairs with |ΔMFE| < 0.5 and |ΔMFE| > 0.5, respectively. **C.–D**. The dot plots of the RNA base pairing for two allele pairs. ΔMFE = 3.3 kCal/Mol (C);ΔMFE = 1.5 kCal/Mol (D). wild-type: major allele; mutant: minor allele. The mutant positions are highlighted in yellow. **E.–F**. The RNA structures corresponding to the wild-type and mutant alleles in (C), respectively. **G.–H**. The RNA structures corresponding to the wild-type and mutant allele in (D), respectively.

Supplementary Figure S11 **Comparisons between ΔMFE and dMFE**. The comparison between ΔMFE derived from our method and dMFE calculated by remuRNA.

Supplementary Figure S12 **Comparisons of the metrics derived by remuRNA and RNAsnp between high and low |*AFCP*| allele pair subgroups**. **A**. Comparison results of the positive group (i.e., *AFCP* > 0.0). **B**. Comparison results of the negative group (i.e., *AFCP* < 0.0). For both positive and negative groups, samples were further divided into two subgroups, i.e., |*AFCP*| > 3 as high |*AFCP*| group and |*AFCP*| < 1.25 as low |*AFCP*| group. *, 10^-5^ < *P* < 0.01; **, 10^-10^ < *P* < 10^-5^; ***, 10^-10^ < *P* < 10^-20^; ****, *P* < 10^-20^; -, 0.01 < *P* < 0.1; --, *P* > 0.1; rank sum test for Delta_MFE, dMFE, P value and P value.1; Student's t test for the rest metrics.

## References

[1] Cohn W.E. (1951). Some results of the applications of ion-exchange chromatography to nucleic acid chemistry. J Cell Physiol Suppl.

[2] Machnicka M.A., Milanowska K., Osman Oglou O., Purta E., Kurkowska M., Olchowik A. (2013). MODOMICS: a database of RNA modification pathways–2013 update. Nucleic Acids Res.

[3] Cohn W.E. (1960). Pseudouridine, a carbon-carbon linked ribonucleoside in ribonucleic acids: isolation, structure, and chemical characteristics. J Biol Chem.

[4] Kierzek E., Malgowska M., Lisowiec J., Turner D.H., Gdaniec Z., Kierzek R. (2013). The contribution of pseudouridine to stabilities and structure of RNAs. Nucleic Acids Res.

[5] Nanda R.K., Tewari R., Govil G., Smith I.C.P. (1974). The conformation of β-pseudouridine about the glycosidic bond as studied by ^1^H homonuclear overhauser measurements and molecular orbital calculations. Can J Chem.

[6] Davis D.R. (1995). Stabilization of RNA stacking by pseudouridine. Nucleic Acids Res.

[7] Arnez J.G., Steitz T.A. (1994). Crystal structure of unmodified tRNA(Gln) complexed with glutaminyl-tRNA synthetase and ATP suggests a possible role for pseudo-uridines in stabilization of RNA structure. Biochemistry.

[8] Jack K., Bellodi C., Landry D.M., Niederer R.O., Meskauskas A., Musalgaonkar S. (2011). rRNA pseudouridylation defects affect ribosomal ligand binding and translational fidelity from yeast to human cells. Mol Cell.

[9] Auffinger P., Westhof E., Grosjean H., Benne R. (1998). Modification and Editing of RNA.

[10] Durant P.C., Davis D.R. (1999). Stabilization of the anticodon stem-loop of tRNA^Lys,3^ by an A^+^-C base-pair and by pseudouridine. J Mol Biol.

[11] Yu A.T., Ge J., Yu Y.T. (2011). Pseudouridines in spliceosomal snRNAs. Protein Cell.

[12] Karijolich J., Yi C., Yu Y.T. (2015). Transcriptome-wide dynamics of RNA pseudouridylation. Nat Rev Mol Cell Biol.

[13] Ganot P., Bortolin M.L., Kiss T. (1997). Site-specific pseudouridine formation in preribosomal RNA is guided by small nucleolar RNAs. Cell.

[14] Hunter S., Jones P., Mitchell A., Apweiler R., Attwood T.K., Bateman A. (2011). InterPro in 2011: new developments in the family and domain prediction database. Nucleic Acids Res.

[15] Lewis C.J.T., Pan T., Kalsotra A. (2017). RNA modifications and structures cooperate to guide RNA–protein interactions. Nat Rev Mol Cell Biol.

[16] Bakin A.V., Ofengand J. (1998). Mapping of pseudouridine residues in RNA to nucleotide resolution. Methods Mol Biol.

[17] Carlile T.M., Rojas-Duran M.F., Gilbert W.V. (2015). Pseudo-Seq: genome-wide detection of pseudouridine modifications in RNA. Methods Enzymol.

[18] Schwartz S., Bernstein D.A., Mumbach M.R., Jovanovic M., Herbst R.H., Léon-Ricardo B.X. (2014). Transcriptome-wide mapping reveals widespread dynamic-regulated pseudouridylation of ncRNA and mRNA. Cell.

[19] Lovejoy AF, Riordan DP, Brown PO. Transcriptome-wide mapping of pseudouridines: pseudouridine synthases modify specific mRNAs in *S. cerevisiae*. PLoS One 2014;9:e110799. 10.1371/journal.pone.0110799 PMC4212993 25353621

[20] Li X., Zhu P., Ma S., Song J., Bai J., Sun F. (2015). Chemical pulldown reveals dynamic pseudouridylation of the mammalian transcriptome. Nat Chem Biol.

[21] Li Y.H., Zhang G., Cui Q. (2015). PPUS: a web server to predict PUS-specific pseudouridine sites. Bioinformatics.

[22] Chen W., Tang H., Ye J., Lin H., Chou K.C. (2016). iRNA-PseU: identifying RNA pseudouridine sites. Mol Ther Nucleic Acids.

[23] Zhang S., Zhou J., Hu H., Gong H., Chen L., Cheng C. (2015). A deep learning framework for modeling structural features of RNA-binding protein targets. Nucleic Acids Res.

[24] Zhou J., Troyanskaya O.G. (2015). Predicting effects of noncoding variants with deep learning-based sequence model. Nat Methods.

[25] Leung M.K.K., Xiong H.Y., Lee L.J., Frey B.J. (2014). Deep learning of the tissue-regulated splicing code. Bioinformatics.

[26] Lecun Y., Bottou L., Bengio Y., Haffner P. (1998). Gradient-based learning applied to document recognition. Proc IEEE.

[27] LeCun Y., Bengio Y., Hinton G. (2015). Deep learning. Nature.

[28] Hinton G.E., Deng L., Yu D., Dahl G.E., Mohamed A., Jaitly N. (2012). Deep neural networks for acoustic modeling in speech recognition: the shared views of four research groups. IEEE Signal Proc Mag.

[29] Collobert R., Weston J., Bottou L., Karlen M., Kavukcuoglu K., Kuksa P. (2011). Natural language processing (almost) from scratch. J Mach Learn Res.

[30] Sun W.J., Li J.H., Liu S., Wu J., Zhou H., Qu L.H. (2015). RMBase: a resource for decoding the landscape of RNA modifications from high-throughput sequencing data. Nucleic Acids Res.

[31] Bergstra J., Bengio Y. (2012). Random search for hyper-parameter optimization. J Mach Learn Res.

[32] He K., Zhang X., Ren S., Sun J. (2015). Delving deep into rectifiers: surpassing human-level performance on ImageNet classification. Proc IEEE Int Conf Comput Vis.

[33] Kelley D.R., Snoek J., Rinn J.L. (2016). Basset: learning the regulatory code of the accessible genome with deep convolutional neural networks. Genome Res.

[34] Weirauch M.T., Yang A., Albu M., Cote A.G., Montenegro-Montero A., Drewe P. (2014). Determination and inference of eukaryotic transcription factor sequence specificity. Cell.

[35] Kulakovskiy I.V., Medvedeva Y.A., Schaefer U., Kasianov A.S., Vorontsov I.E., Bajic V.B. (2012). HOCOMOCO: a comprehensive collection of human transcription factor binding sites models. Nucleic Acids Res.

[36] Gupta S., Stamatoyannopoulos J.A., Bailey T.L., Noble W. (2007). Quantifying similarity between motifs. Genome Biol.

[37] Medina-Rivera A., Defrance M., Sand O., Herrmann C., Castro-Mondragon J.A., Delerce J. (2015). RSAT 2015: regulatory sequence analysis tools. Nucleic Acids Res.

[38] Thomsen M.C.F., Nielsen M. (2012). Seq2Logo: a method for construction and visualization of amino acid binding motifs and sequence profiles including sequence weighting, pseudo counts and two-sided representation of amino acid enrichment and depletion. Nucleic Acids Res.

[39] Ghandi M., Lee D., Mohammad-Noori M., Beer M.A. (2014). Enhanced regulatory sequence prediction using gapped *k*-mer features. PLoS Comput Biol.

[40] Carlile T.M., Rojas-Duran M.F., Zinshteyn B., Shin H., Bartoli K.M., Gilbert W.V. (2014). Pseudouridine profiling reveals regulated mRNA pseudouridylation in yeast and human cells. Nature.

[41] Xie J., Zhang M., Zhou T., Hua X., Tang L., Wu W. (2007). Sno/scaRNAbase: a curated database for small nucleolar RNAs and cajal body-specific RNAs. Nucleic Acids Res.

[42] Zhang T., Huang X.H., Dong L., Hu D., Ge C., Zhan Y.Q. (2010). PCBP-1 regulates alternative splicing of the *CD44* gene and inhibits invasion in human hepatoma cell line HepG2 cells. Mol Cancer.

[43] Das T.P., Suman S., Alatassi H., Ankem M.K., Damodaran C. (2016). Inhibition of AKT promotes FOXO3a-dependent apoptosis in prostate cancer. Cell Death Dis.

[44] Chen C., Zhao X., Kierzek R., Yu Y.T. (2010). A flexible RNA backbone within the polypyrimidine tract is required for U2AF^65^ binding and pre-mRNA splicing *in vivo*. Mol Cell Biol.

[45] Bentley D.L. (2014). Coupling mRNA processing with transcription in time and space. Nat Rev Genet.

[46] Hesketh J. 3′ UTRs and regulation. eLS 2005; https://doi.org/10.1038/npg.els.0005011.

[47] Mignone F, Pesole G. mRNA untranslated regions (UTRs). eLS 2011; https://doi.org/10.1002/9780470015902.a0005009.pub3.

[48] Maden B.E. (1990). The numerous modified nucleotides in eukaryotic ribosomal RNA. Prog Nucleic Acid Res Mol Biol.

[49] Liang X.H., Liu Q., Fournier M.J. (2007). rRNA modifications in an intersubunit bridge of the ribosome strongly affect both ribosome biogenesis and activity. Mol Cell.

[50] King T.H., Liu B., McCully R.R., Fournier M.J. (2003). Ribosome structure and activity are altered in cells lacking snoRNPs that form pseudouridines in the peptidyl transferase center. Mol Cell.

[51] Lorenz R, Bernhart SH, H¨oner Zu Siederdissen C, Tafer H, Flamm C, Stadler PF, et al. ViennaRNA package 2.0. Algorithms Mol Biol 2011;6:26. 10.1186/1748-7188-6-26 PMC3319429 22115189

[52] Lowe T.M., Eddy S.R. (1997). tRNAscan–SE: a program for improved detection of transfer RNA genes in genomic sequence. Nucleic Acids Res.

[53] Kariko K., Muramatsu H., Welsh F.A., Ludwig J., Kato H., Akira S. (2008). Incorporation of pseudouridine into mRNA yields superior nonimmunogenic vector with increased translational capacity and biological stability. Mol Ther.

[54] Mishima Y., Tomari Y. (2016). Codon usage and 3′ UTR length determine maternal mRNA stability in zebrafish. Mol Cell.

[55] Imamachi N., Salam K.A., Suzuki Y., Akimitsu N. (2016). A GC-rich sequence feature in the 3′ UTR directs UPF1-dependent mRNA decay in mammalian cells. Genome Res.

[56] Karijolich J., Yu Y.T. (2011). Converting nonsense codons into sense codons by targeted pseudouridylation. Nature.

[57] dos Reis M., Savva R., Wernisch L. (2004). Solving the riddle of codon usage preferences: a test for translational selection. Nucleic Acids Res.

[58] Sharp P.M., Li W.H. (1987). The codon adaptation index—a measure of directional synonymous codon usage bias, and its potential applications. Nucleic Acids Res.

[59] Clarke T.F., Clark P.L. (2008). Rare codons cluster. PLoS One.

[60] Gao X., Wan J., Liu B., Ma M., Shen B., Qian S.B. (2014). Quantitative profiling of initiating ribosomes *in vivo*. Nat Methods.

[61] Shen L.X., Basilion J.P., Stanton V.P. (1999). Single-nucleotide polymorphisms can cause different structural folds of mRNA. Proc Natl Acad Sci U S A.

[62] Johnson AD, Trumbower H, Sadee W. RNA structures affected by single nucleotide polymorphisms in transcribed regions of the human genome. WebmedCentral Bioinformatics 2011;2:WMC001600.

[63] Haas U., Sczakiel G., Laufer S. (2012). MicroRNA-mediated regulation of gene expression is affected by disease-associated SNPs within the 3′ UTR via altered RNA structure. RNA Biol.

[64] Salari R., Kimchi-Sarfaty C., Gottesman M.M., Przytycka T.M. (2012). Sensitive measurement of single-nucleotide polymorphism-induced changes of RNA conformation: application to disease studies. Nucleic Acids Res.

[65] Sabarinathan R., Tafer H., Seemann S.E., Hofacker I.L., Stadler P.F., Gorodkin J. (2013). RNAsnp: efficient detection of local RNA secondary structure changes induced by SNPs. Human Mutat.

[66] Armstrong D.L., Zidovetzki R., Alarcón-Riquelme M.E., Tsao B.P., Criswell L.A., Kimberly R.P. (2014). GWAS identifies novel SLE susceptibility genes and explains the association of the HLA region. Genes Immun.

[67] Lauc G., Huffman J.E., Pucic M., Zgaga L., Adamczyk B., Muzinic A. (2013). Loci associated with *N*-glycosylation of human immunoglobulin G show pleiotropy with autoimmune diseases and haematological cancers. PLoS Genet.

[68] Hershberg R., Petrov D.A. (2008). Selection on codon bias. Ann Rev Genet.

[69] Quax T.E.F., Claassens N.J., Söll D., van der Oost J. (2015). Codon bias as a means to fine-tune gene expression. Mol Cell.

[70] Purvis I.J., Bettany A.J., Santiago T.C., Coggins J.R., Duncan K., Eason R. (1987). The efficiency of folding of some proteins is increased by controlled rates of translation *in vivo*: a hypothesis. J Mol Biol.

[71] Presnyak V., Alhusaini N., Chen Y.H., Martin S., Morris N., Kline N. (2015). Codon optimality is a major determinant of mRNA stability. Cell.

[72] Wang X., Zhao B.S., Roundtree I.A., Lu Z., Han D., Ma H. (2015). *N*^6^-methyladenosine modulates messenger RNA translation efficiency. Cell.

[73] Ruiz A., Heilmann S., Becker T., Hernandez I., Wagner H., Thelen M. (2014). Follow-up of loci from the international genomics of Alzheimer’s disease project identifies *TRIP4* as a novel susceptibility gene. Transl Psychiatry.

