# Supplementary material for: Characterizing RNA Pseudouridylation by Convolutional Neural Networks
Source: Genomics Proteomics Bioinformatics. 2021 Feb 23;19(5):815–33. doi: 10.1016/j.gpb.2019.11.015 (PMC9170758; doi:10.1016/j.gpb.2019.11.015)
Supplement: Supplementary File S1 — Supplementary descriptions of methods and results (in PDF format). [file mmc1.rtf]

File S1  Supplementary descriptions of methods and results (in PDF format)

Supplementary results
Additional validation of the PULSE model
To further validate the prediction accuracy of PULSE, we also tested it on three small but relatively more reliable datasets of pseudouridine sites (Table S1 and Table S2), including a dataset of high-fidelity pseudouridine sites that were identified by the SCARLET (site-specific cleavage and radioactive-labeling followed by ligation-assisted extraction and thin-layer chromatography) technique [1], and two datasets of the conserved pseudouridine sites for both human and mouse [2]. In these new validation tests, all overlapping sites were removed from the training data. Our validation tests on these three additional datasets of high-fidelity pseudouridine sites showed that PULSE can still achieve high classification accuracy, with the AUC scores between 0.86–0.91 (Table 1). We also compared PULSE to the previous methods (i.e., PPUS and iRNAPseU) on these three datasets, the results showed that our models performed better.

Table 1  Validation and comparison results
Dataset	Predictor	Specificity	Sensitivity	Accuracy	AUC	
Conserved data (Human)	hPULSE	0.77	0.77	0.77	0.91	
	iRNAPseU	0.77	0.77	0.77	-	
	PPUS	1.00	0.38	0.69	-	
Conserved data (Mouse)	mPULSE	0.77	0.83	0.80	0.87	
	iRNAPseU	0.62	0.58	0.60	-	
SCARLET data	hPULSE	0.67	0.83	0.75	0.86	
	iRNAPseU	0.50	0.83	0.67	-	
	PPUS	1.00	0.00	0.50	-	

The validation and comparison results on the conserved Ø sites across human and mouse [2] and a set of the pseudouridine sites in human [2] that were identified by the SCARLET (site-specific cleavage and radioactive-labeling followed by ligation-assisted extraction and thin-layer chromatography) technique [1]. During the evaluation process, for both hPULSE and mPULSE models, the overlapping pseudouridine sites were removed from training data. The AUC results of PPUS and iRNAPseU were not showed here because they were in binary format.
In addition to the baseline approach gkmSVM, we also compared the prediction performance to that of current available tools for predicting pseudouridine sites, including PPUS [3] and iRNA-PseU [4]. The comparison tests on both human and mouse datasets via 10-fold cross validation demonstrated that PULSE significantly outperform these two state-of-the-art methods, with an increase of accuracy by 8%–22% (Table 2).

Table 2  Comparisons of prediction performance between PULSE and previous methods
Species	Predictor	Acc	MCC	Sn	Sp	
Human	hPULSE	0.77	0.55	0.80	0.74	
	iRNAPseU	0.65	0.30	0.60	0.69	
	PPUS	0.55	0.23	0.10	1.00	
Mouse	mPULSE	0.77	0.54	0.74	0.80	
	iRNAPseU	0.69	0.38	0.74	0.64	
Note: This table shows the comparison results of prediction performance between PULSE and previous methods (i.e., PPUS and iRNA-PseU). The prediction results of PPUS on the mouse dataset are not shown here, as the PPUS server does not provide such an option. Acc, accuracy; MCC, Matthews correlation coefficient; Sn, sensitivity; Sp, specificity.

Clustering of motifs captured by PULSE
After the sequence motifs were extracted by the filters of the first convolution layer, we used the RSAT tool [5] to cluster all these sequence patterns identified by PULSE and obtained 70 and 69 clusters for human and mouse, respectively (Figure S2; Materials and methods). Based on the consensus sequence features of each cluster, more knowledge about pseudouridylation can be obtained, such as the proteins whose bindings are required for the pseudouridylation process. In addition, the clusters of sequence features can imply the subtypes of pseudouridylation, which may provide novel insights into understanding the underlying mechanisms of pseudouridylation.

Additional analysis results on the relationships between pseudouridylation and RNA stability
To further verify the relationships between pseudouridylation and RNA stability, we performed additional analyses on several other curated RNA half-life datasets obtained from the literatures [6,7]. In particular, we collected additional RNA half-life datasets from seven lymphoblastoid cell lines (GM07019, GM07029, GM10835, GM12812, GM12813, GM12814, and GM12815) [6] and one mouse Neuro-2a cell line [7]. We found that seven of these eight datasets displayed a similar trend to our previous analysis result, while only one (GM12815) showed an insignificant difference (Figure S9). These additional analysis results suggested that the relationship that we previously claimed between pseudouridylation and RNA stability was probably robust to different cell lines.

Supplementary methods
Feature encoding of the sequence profiles
We use the following scheme to encode individual nucleotides in a given input sequence S,
A = [1., 0., 0., 0.],
T = [0., 1., 0., 0.],
G = [0., 0., 1., 0.],
C = [0., 0., 0., 1.],
N = [0.25, 0.25, 0.25, 0.25]
where 'N' stands for any nucleotide. Then a given sequence can be encoded into a matrix. For example, sequence S = 'ATGCAN' can be encoded as
.

Basics of CNNs
Our computational pipeline PULSE is composed of two alternate convolution and pooling layers, which are then stacked with a fully-connected neural network consisting of three hidden layers. More specifically, given an RNA sequence of length L and the corresponding feature-encoding matrix S, the convolution result  is an  array, and can be written as
,
where n and mc stand for the number and the size of convolution operators, respectively, ,  and W represents the weight matrix of the n convolution operators, i.e.,  represents the value at the j-th row and the t-th column of the k-th kernel. In the rectification stage, the parametric rectified linear activation function (PReLU) [8] is applied to the previous convolved result X, that is,

where  stands for the slope of the negative part which can be learned in the model training process.
In the pooling stage, the dimension of Y is reduced by a max-pooling operator of size . Suppose that the pooling result is defined as Z = max_pool(Y). Then we have
,
where  stands for the index of the pooling operator and j stands for the start position of pooling. 
Next, the output matrix Z of max-pooling is PReLU rectified, flattened to a high-dimensional vector and then fed to a fully-connected neural network, which can be formulated as , where  represents the weight matrix of the fully-connected network. For final classification, a softmax layer is employed in the last layer of the fully-connected network, that is,
,
where  stands for the weight matrix of the softmax layer,  stands for the output of the proceeding layer in the fully-connected network, and K stands for the length of .

The GO enrichment analysis procedure
Gene ontology (GO) enrichment analysis can highlight the consensus functions of a particular gene set, which may help reveal the latent features of the genes in the set [9]. For gene ontology (GO) analysis, we first selected top 500 mRNA transcripts with the highest tPPS values. Then we uploaded the gene names of these 500 transcripts to DAVID [10,11], and ran the functional annotation clustering module with the default parameters for GO enrichment analysis. During the analysis, the P values were calculated based on the binomial distribution and the Benjamini corrected P values were used for selecting out the final significant GO terms.

The procedure for analyzing the SNV associations
We mainly considered those single-nucleotide variants (SNVs) with the risk alleles from the known genome- wide association studies (GWAS). In particular, we first downloaded the GWAS annotation data from the GWAS Catalog of EMBL-EBI [12] (i.e., the file 'All associations with added ontology annotations'). Then we searched the SNP (single-nucleotide polymorphism) database UCSC SNP147 [13] to obtain the genomic coordinates of the downloaded GWAS SNVs. Next, we screened out those SNVs that were validated by 1000Genomes [14] for the downstream analyses. After that, we applied PULSE to predict the lPPS value of the thymine position that was closest to each of the selected GWAS SNVs. We computed the lPPS values for the sequences of both major and minor alleles, and then defined a metric, called the allele fold-change of pseudouridylation potential (AFCP), which was defined by
,
where  and  represent the lPPS values of the major and the corresponding minor alleles, respectively. 
We used RNAfold [15] to estimate the minimum free energy (MFE) of the input sequence (which had a length of 101 nts) centered at the thymine site of interest. Then the difference of MFE between major and minor alleles were calculated as,
,
where Ema and Emi stand for the MFE values of the major and the corresponding minor alleles, respectively. 
To further investigate the relationships between pseudouridylation and the effects of SNVs on RNA structures, we also applied remuRNA and RNAsnp to predict an esemble of RNA secondary structures and performed the similar analyses. Both remuRNA and RNAsnp were downloaded from https://github.com/ mmiladi/RNA-SNV-wrapper. The dot plots of the allele pairs showed in supplementary figures were derived from the RNAsnp web server [16] (https://rth.dk/resources/rnasnp/).

Calculation of codon usage bias
We computed the tAI values of codons based on the tRNA gene copy numbers and the codon-anticodon pairing strength according to the Crick wobble rule [17] using the following expressions,
,
,
where Wi stands for the absolute tRNA adaptation index of codon i, ni stands for the number of tRNAs that can be matched to codon i, sij stands for the coefficient of the codon-anticodon pairing strength between codon i and its j-th tRNA, tGCNij stands for the gene copy number of the j-th tRNA of codon i, and wi represents the final normalized tAI value. For all the wobble pairs, including I:U, G:C, U:A, C:G, G:U, I:C, I:A, U:G, L:A, the corresponding coefficient vector of sij used in our analysis was [0, 0, 0, 0, 0.41, 0.28, 0.9999, 0.68, 0.89], which was adapted from [17].
The CAI value of a given codon was computed mainly based on its usage frequency [18]. More specifically, for codon i, its CAI value Ci is calculated by
,
,
where ni stands for the number of synonymous codons of codon i and wi stands for the usage frequency of codon i.
The %MinMax profiles of codons were calculated according to [19]. In particular, for the i-th synonymous codon of the j-th codon position that covers N codons (here N was set to 21 in our study) in a coding sequence, its actual codon usage frequency and the average codon usage frequency, denoted as Fij and Favg,j, respectively, can be computed as follows,
,
,
,
where nj stands for the total number of synonymous codons of the j-th codon position in the coding sequence. Then the %MinMax value of the j-th codon position in that sequence can be calculated as,


The procedure for analyzing the association between the predicted pseudouridylation potentials and mRNA stability
The sequences of the 3' UTR and CDS regions of the transcripts with known half-life information were downloaded from Ensembl [20] using BioMart [21]. In our analysis, we mainly used the RNA half-life data that were previously curated from [22], which contained 7475 transcripts in total. We then used PULSE to compute the tPPS profiles of the 3' UTR and CDS regions of these genes with known half-life information. Next, we divided these genes into two groups according to whether the tPPS values of their 3' UTR or CDS regions were larger than the average level or not. After that, we compared the half-lives of the genes between these two groups and investigated the possible relation between pseudouridylation and mRNA half-lives. We also examined the potential relation between the lengths and the GC-contents of 3' UTRs and their predicted pseudouridylation potentials by performing the similar analysis.

The procedure for analyzing ribosome profiling data
The ribosome profiling data of human cell line HEK293 (collected from [23]), including the ribosome profiles of both translation initiation and elongation, were downloaded from GWIPS-viz [24] using its table browser. We first normalized the ribosome profiling data with respect to their corresponding mRNA coverages. Then we mapped the normalized ribosome profiles to genes according to their genomic positions and thus obtained the intragenic ribosome profiles. Next, we applied PULSE to predict the lPPS values of those thymine positions that were located within the +/- 1 codon range of each intragenic genomic position. After that, we extracted two groups of codons from these selected thymine positions according to their predicted lPPS values, which had the lPPS values greater than 75% quantile (termed by '> 3rd Qu.') and lower than 25% quantile (termed by '< 1st Qu.'), respectively. Finally, we compared the ribosome profiles of codons in these two groups and investigated the relations between the predicted lPPS values and the corresponding ribosome footprints during the translation process.

References
[1] Nian Liu, Marc Parisien, Qing Dai, Guanqun Zheng, Chuan He, and Tao Pan. Probing N6-methyladenosine RNA modification status at single nucleotide resolution in mRNA and long noncoding RNA. RNA (New York, N.Y.), 19:1848–1856, Dec 2013.
[2] Xiaoyu Li, Ping Zhu, Shiqing Ma, Jinghui Song, Jinyi Bai, Fangfang Sun, and Chengqi Yi. Chemical pulldown reveals dynamic pseudouridylation of the mammalian transcriptome. Nature Chemical Biology, 11(8):592–597, jun 2015.
[3] Yan-Hui Li, Gaigai Zhang, and Qinghua Cui. PPUS: a web server to predict PUS-specific pseudouridine sites. Bioinformatics, 31(20):3362–3364, jun 2015.
[4] Wei Chen, Hua Tang, Jing Ye, Hao Lin, and Kuo-Chen Chou. iRNA-PseU: Identifying RNA pseudouridine sites. Molecular Therapy-Nucleic Acids, 5(7):e332, 2016.
[5] Alejandra Medina-Rivera, Matthieu Defrance, Olivier Sand, Carl Herrmann, Jaime A Castro-Mondragon, Jeremy Delerce, Sébastien Jaeger, Christophe Blanchet, Pierre Vincens, Christophe Caron, Daniel M Staines, Bruno Contreras-Moreira, Marie Artufel, Lucie Charbonnier-Khamvongsa, Céline Hernandez, Denis Thieffry, Morgane Thomas-Chollier, and Jacques van Helden. RSAT 2015: Regulatory sequence analysis tools. Nucleic acids research, 43:W50–W56, Jul 2015.
[6] Jubao Duan, Jianxin Shi, Xijin Ge, Lars Dölken, Winton Moy, Deli He, Sandra Shi, Alan R. Sanders, Jeff Ross, and Pablo V. Gejman. Genome-wide survey of interindividual differences of RNA stability in human lymphoblastoid cell lines. Sci. Rep., 3, feb 2013.
[7] Michael B Clark, Rebecca L Johnston, Mario Inostroza-Ponta, Archa H Fox, Ellen Fortini, Pablo Moscato, Marcel E Dinger, and John S Mattick. Genome-wide analysis of long noncoding RNA stability. Genome research, 22:885–898, May 2012.
[8] K. He, X. Zhang, S. Ren, and J. Sun. Delving deep into rectifiers: Surpassing human-level performance on ImageNet classification. In Proc. IEEE Int. Conf. Computer Vision (ICCV), pages 1026–1034, December 2015.
[9] Michael Ashburner, Catherine A. Ball, Judith A. Blake, David Botstein, Heather Butler, J. Michael Cherry, Allan P. Davis, Kara Dolinski, Selina S. Dwight, Janan T. Eppig, and et al. Gene ontology: tool for the unification of biology. Nature Genetics, 25(1):25–29, May 2000.
[10] Da Wei Huang, Brad T Sherman, and Richard A Lempicki. Systematic and integrative analysis of large gene lists using DAVID bioinformatics resources. Nature protocols, 4:44–57, 2009.
[11] Da Wei Huang, Brad T Sherman, and Richard A Lempicki. Bioinformatics enrichment tools: paths toward the comprehensive functional analysis of large gene lists. Nucleic acids research, 37:1–13, Jan 2009.
[12] Danielle Welter, Jacqueline MacArthur, Joannella Morales, Tony Burdett, Peggy Hall, Heather Junkins, Alan Klemm, Paul Flicek, Teri Manolio, Lucia Hindorff, and et al. The NHGRI GWAS catalog, a curated resource of SNP-trait associations. Nucleic Acids Research, 42(D1):D1001–D1006, Dec 2013.
[13] K. R. Rosenbloom, J. Armstrong, G. P. Barber, J. Casper, H. Clawson, M. Diekhans, T. R. Dreszer, P. A. Fujita, L. Guruvadoo, M. Haeussler, and et al. The UCSC genome browser database: 2015 update. Nucleic Acids Research, 43(D1):D670–D681, Nov 2014.
[14] Adam Auton, Gon¸calo R. Abecasis, David M. Altshuler, Richard M. Durbin, Gon¸calo R. Abecasis, David R. Bentley, Aravinda Chakravarti, Andrew G. Clark, Peter Donnelly, Evan E. Eichler, and et al. A global reference for human genetic variation. Nature, 526(7571):68–74, Sep 2015.
[15] Ronny Lorenz, Stephan H Bernhart, Christian Höner Zu Siederdissen, Hakim Tafer, Christoph Flamm, Peter F Stadler, and Ivo L Hofacker. ViennaRNA package 2.0. Algorithms for molecular biology : AMB, 6:26, 2011.
[16] Radhakrishnan Sabarinathan, Hakim Tafer, Stefan E Seemann, Ivo L Hofacker, Peter F Stadler, and Jan Gorodkin. Rnasnp: efficient detection of local rna secondary structure changes induced by snps. Human mutation, 34(4):546–556, 2013.
[17] M. d. Reis. Solving the riddle of codon usage preferences: a test for translational selection. Nucleic Acids Research, 32(17):5036–5044, sep 2004.
[18] Paul M. Sharp and Wen-Hsiung Li. The codon adaptation index-a measure of directional synonymous codon usage bias, and its potential applications. Nucl Acids Res, 15(3):1281–1295, 1987.
[19] Thomas F. Clarke and Patricia L. Clark. Rare codons cluster. PLoS ONE, 3(10):e3412, Oct 2008.
[20] Bronwen L. Aken, Sarah Ayling, Daniel Barrell, Laura Clarke, Valery Curwen, Susan Fairley, Julio Fernandez Banet, Konstantinos Billis, Carlos García Girón, Thibaut Hourlier, and et al. The Ensembl gene annotation system. Database, 2016:baw093, 2016.
[21] R. J. Kinsella, A. Kahari, S. Haider, J. Zamora, G. Proctor, G. Spudich, J. Almeida-King, D. Staines, P. Derwent, A. Kerhornou, and et al. Ensembl BioMarts: a hub for data retrieval across taxonomic space. Database, 2011(0):bar030–bar030, Jul 2011.
[22] Lea H. Gregersen, Markus Schueler, Mathias Munschauer, Guido Mastrobuoni, Wei Chen, Stefan Kempa, Christoph Dieterich, and Markus Landthaler. MOV10 is a 5' to 3' RNA helicase contributing to UPF1 mRNA target degradation by translocation along 3' UTRs. Molecular Cell, 54(4):573–585, May 2014.
[23] Xiangwei Gao, Ji Wan, Botao Liu, Ming Ma, Ben Shen, and Shu-Bing Qian. Quantitative profiling of initiating ribosomes in vivo. Nature Methods, 12(2):147–153, dec 2014.
[24] Audrey M. Michel, Gearoid Fox, Anmol M. Kiran, Christof De Bo, Patrick B. F. O'Connor, Stephen M. Heaphy, James P. A. Mullan, Claire A. Donohue, Desmond G. Higgins, and Pavel V. Baranov. GWIPS-viz: development of a ribo-seq genome browser. Nucleic Acids Research, 42(D1):D859–D864, oct 2013.
