# Supplementary figures and images for: Characterizing RNA Pseudouridylation by Convolutional Neural Networks
Source: Genomics Proteomics Bioinformatics. 2021 Feb 23;19(5):815–33. doi: 10.1016/j.gpb.2019.11.015 (PMC9170758; doi:10.1016/j.gpb.2019.11.015)

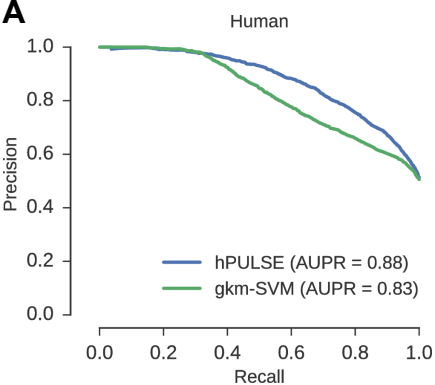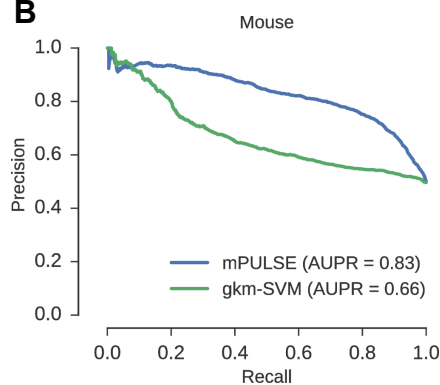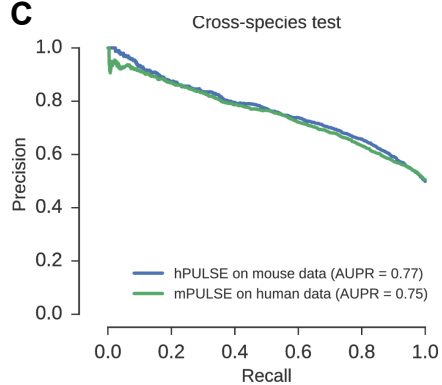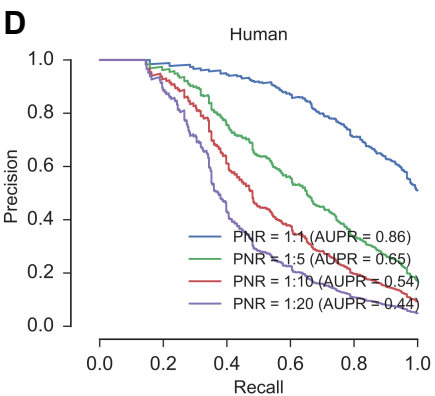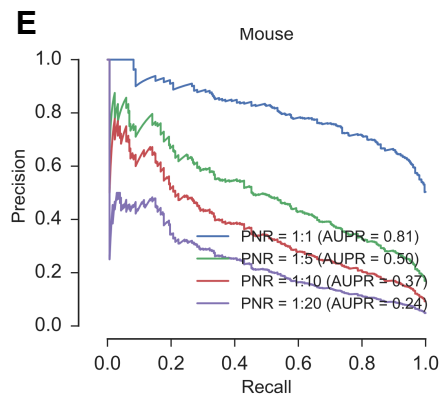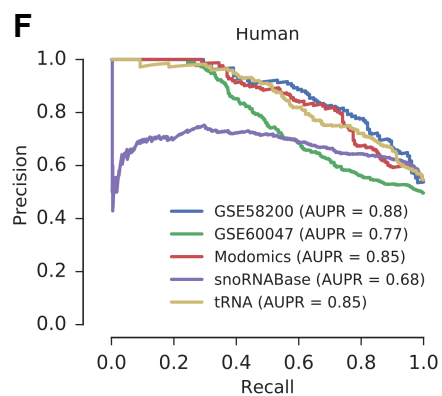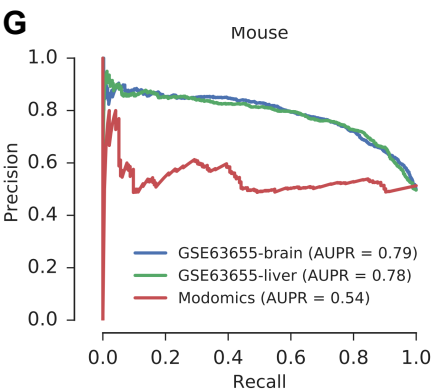

Supplement: Supplementary Figure S1 — The precision-recall (PR) curves and the corresponding area under the precision-recall curve (AUPR) scores in the cross-validation results. A. and B. Comparisons of the 10-fold cross-validation results between PULSE and the baseline approach gkm-SVM for human and mouse, respectively. C. The results on cross-species tests between human and mouse. D. and E. The precision-recall curves and the corresponding AUPR scores of the models on the independent imbalanced datasets with different positive-to-negative ratios (PNRs; including 1:1, 1:5, 1:10, and 1:20) that did not have any overlap with training data. F. and G. The precision-recall curves and the corresponding AUPR scores of the retrained models on individual held-out datasets for human and mouse, respectively. The legends are the same as in Figure 2. [file mmc2.pdf]

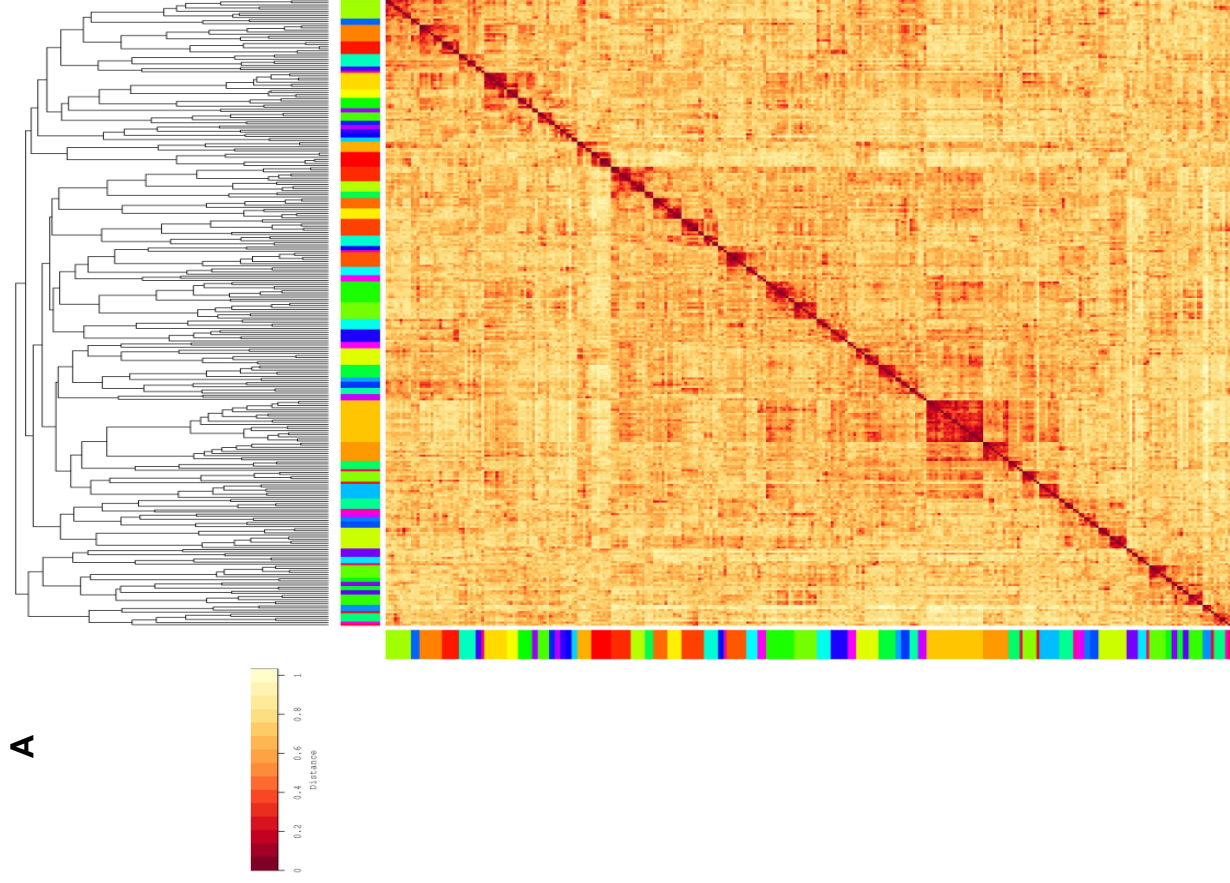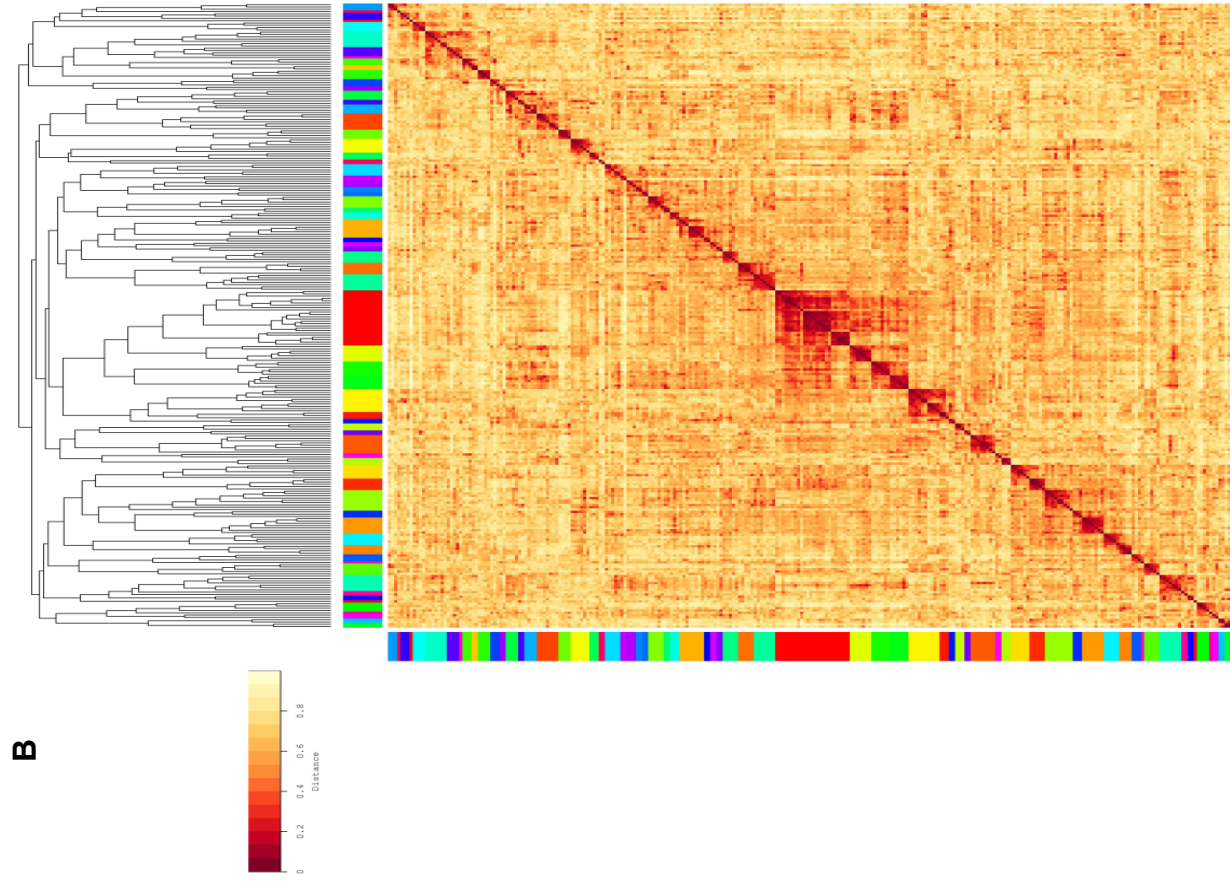

Supplement: Supplementary Figure S2 — Sequence motif clustering. A. and B. The clustering heatmaps of the sequence motifs of pseudouridylation identified by PULSE for human (A) and mouse (B). The sequence motifs of human and mouse had 70 and 69 clusters, respectively. The clusters are labeled by different colors. [file mmc3.pdf]

**A**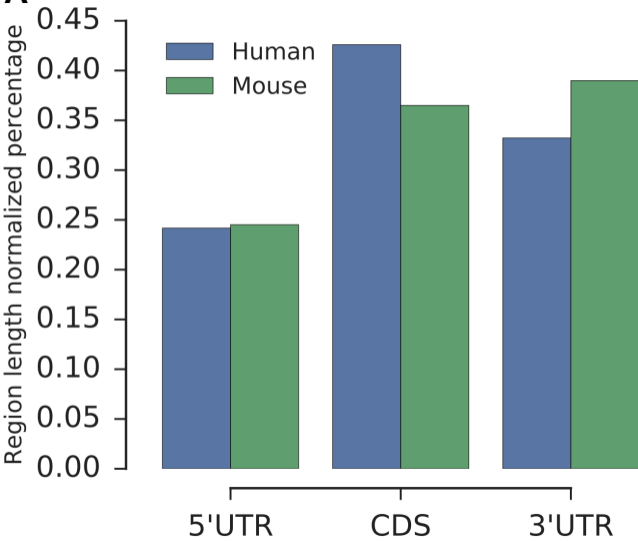**B**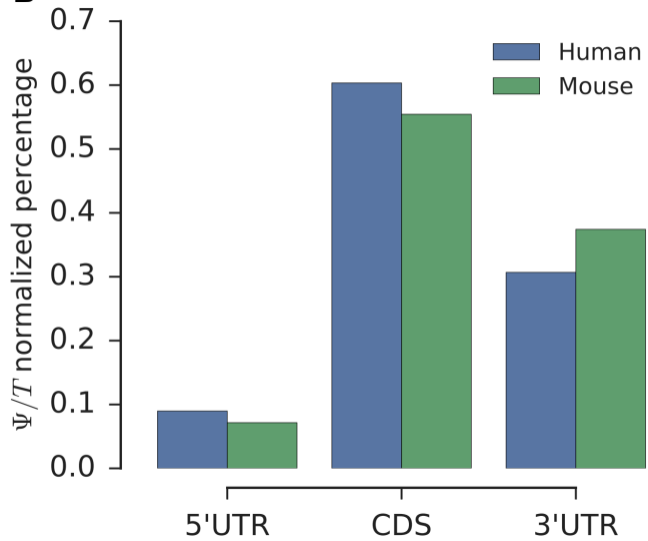

Supplement: Supplementary Figure S3 — The normalized distributions of the predicted Ψ sites in different genomic regions. A. and B. The proportions of the predicted Ψ sites were normalized by the lengths of individual regions and their Ψ/T ratios, respectively. [file mmc4.pdf]

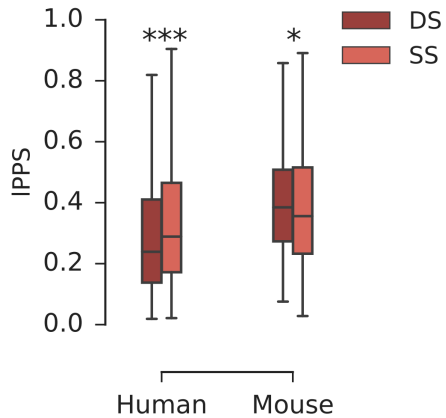

Supplement: Supplementary Figure S4 — Biased distributed pseudouridines in tRNA structures. Comparisons of the lPPS scores computed by PULSE in tRNAs between single-strand (SS) and double-strand (DS) regions. *, 10-10 < P < 10-5; ***, P < 10-30, rank-sum test. [file mmc5.pdf]

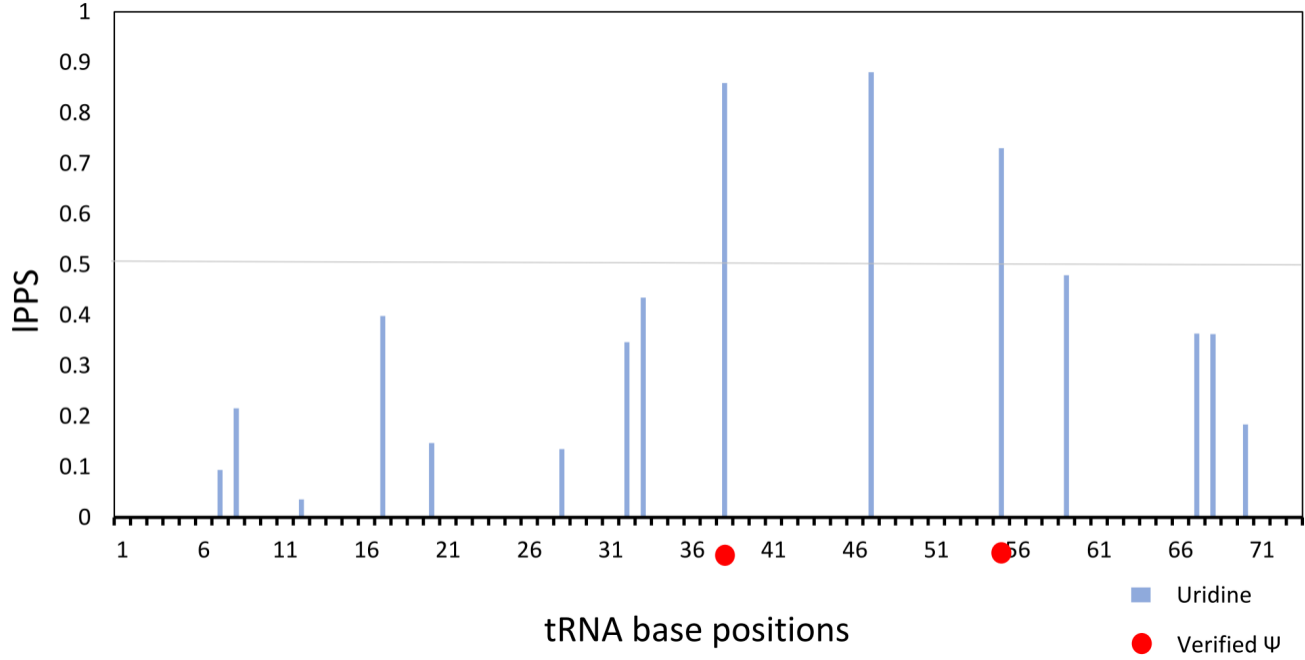

Supplement: Supplementary Figure S5 — Known and novel pseudouridylation sites detected in a tRNA. PULSE accurately detected two experimentally reported pseudouridylation sites (marked by red dots) and one novel site from all 15 uridine candidates (all the three detected sites had lPPS scores above 0.5) in a tRNA (tRNAdb ID: tdbR00000017) corresponding to alanine. [file mmc6.pdf]

**A**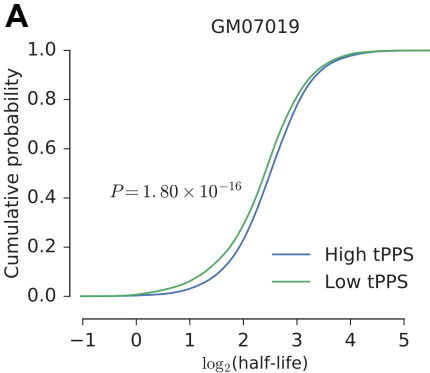**B**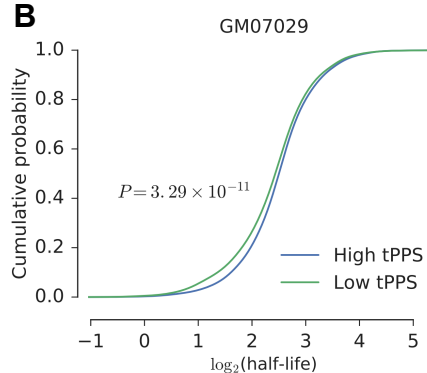**C**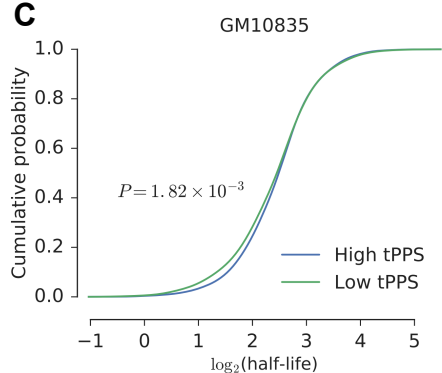**D**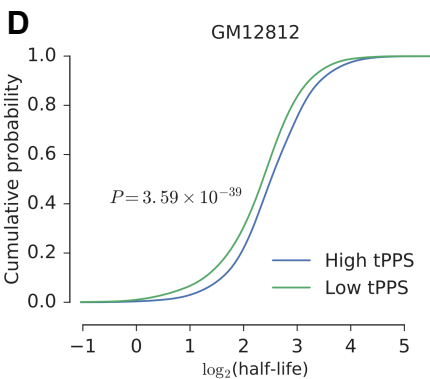**E**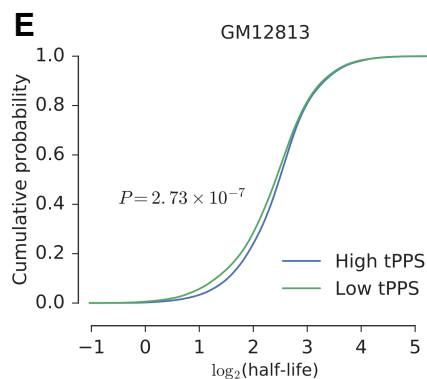**F**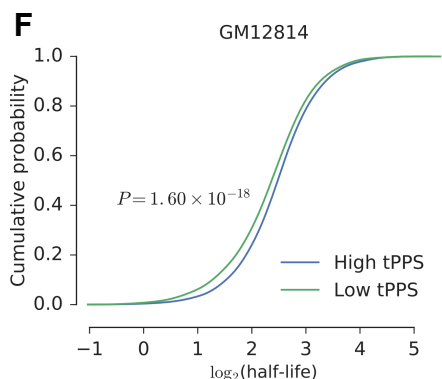**G**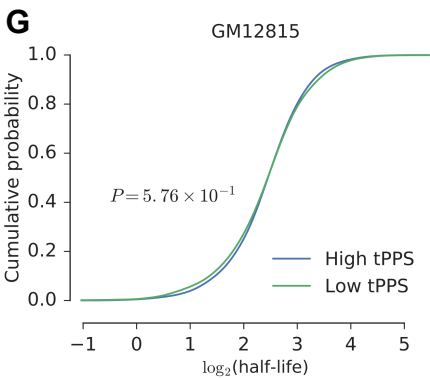**H**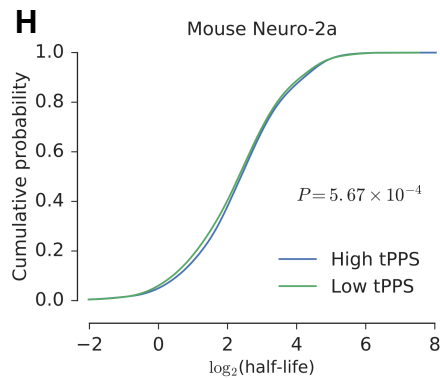

Supplement: Supplementary Figure S6 — Relationships between RNA half-lives and pseudouridylation. Relationships between RNA half-lives and pseudouridylation of seven human lymphoblastoid cell lines (GM07019, GM07029, GM10835, GM12812, GM12813, GM12814, and GM12815) (A–G) and the mouse Neuro-2a cell line (H). [file mmc7.pdf]

**A**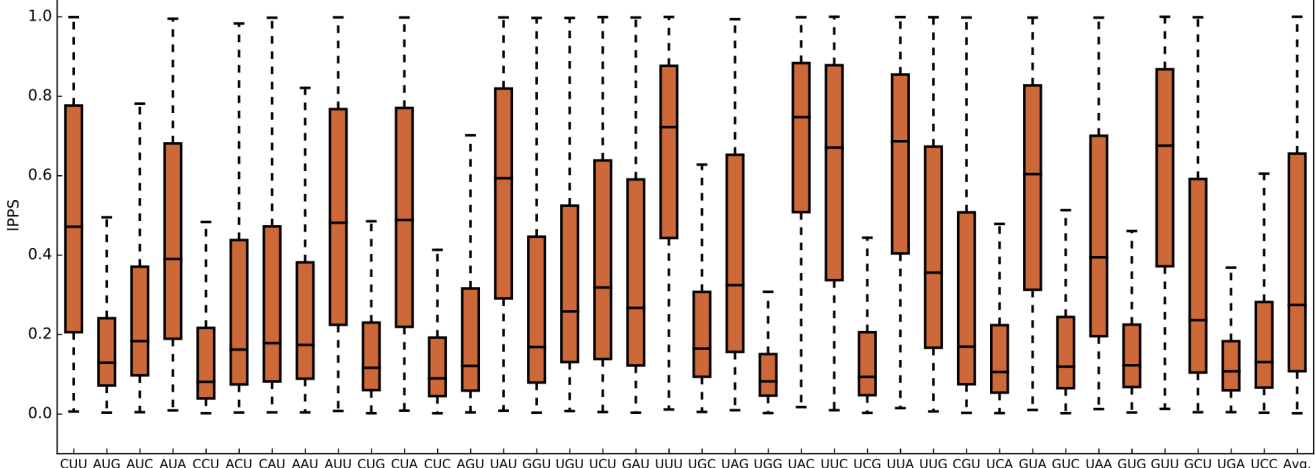**B**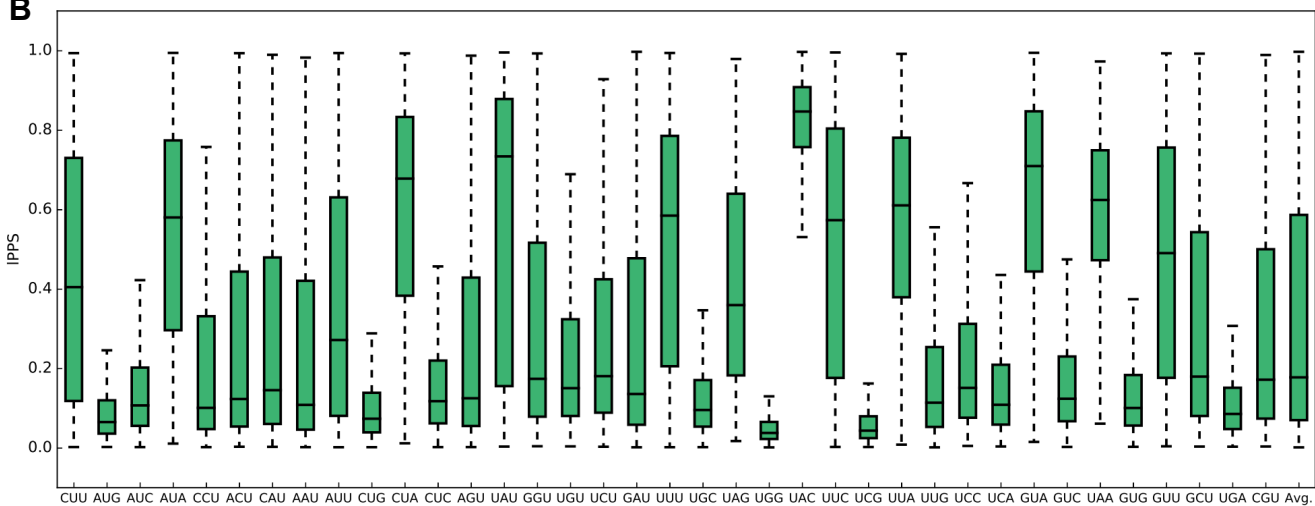

Supplement: Supplementary Figure S7 — Pseudouridine potentials of all uridine-containing condons. The lPPS values predicted by PULSE for all uridine-containing codons for both human (A) and mouse (B). The average lPPS value (termed by ‘Avg’) is also shown. [file mmc8.pdf]

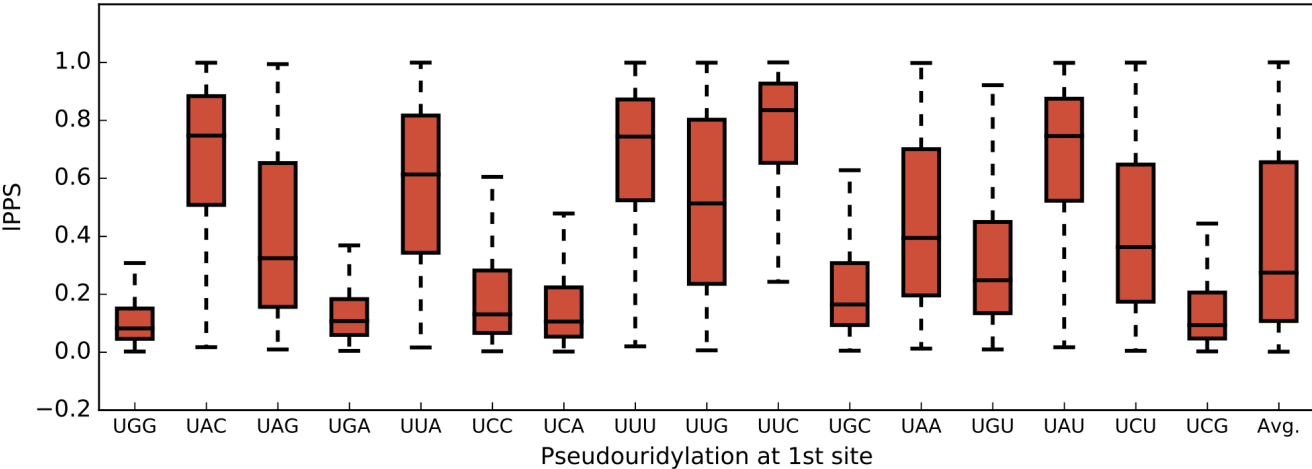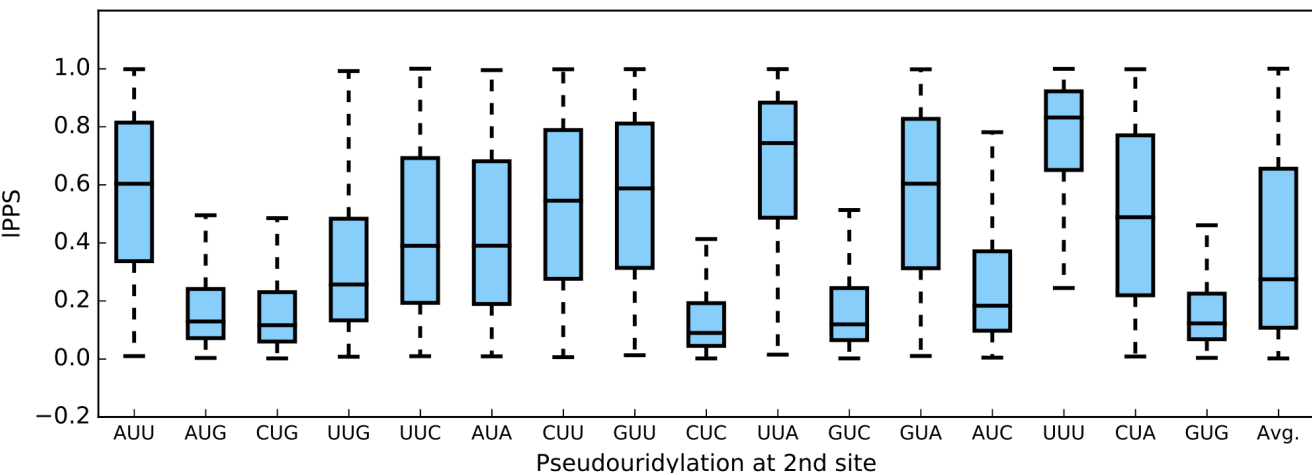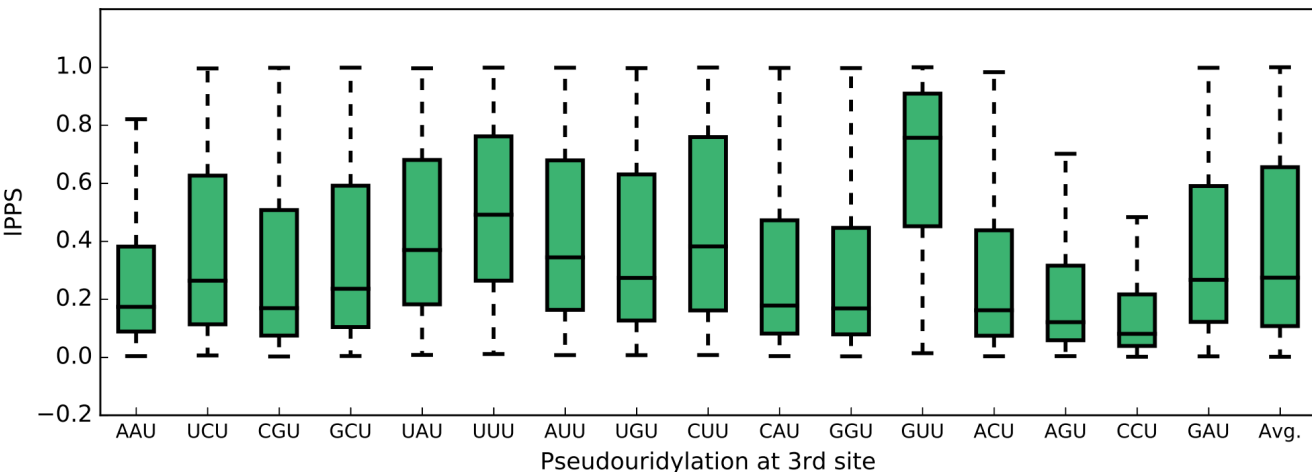

Supplement: Supplementary Figure S8 — Pseudouridine potentials of human uridine-containing codons with uridine in different nucleotide positions. The lPPS values predicted by PULSE for the uridine-containing codons with pseudouridylation in different nucleotide positions for human. The average lPPS value (termed by ‘Avg’) is also shown. [file mmc9.pdf]

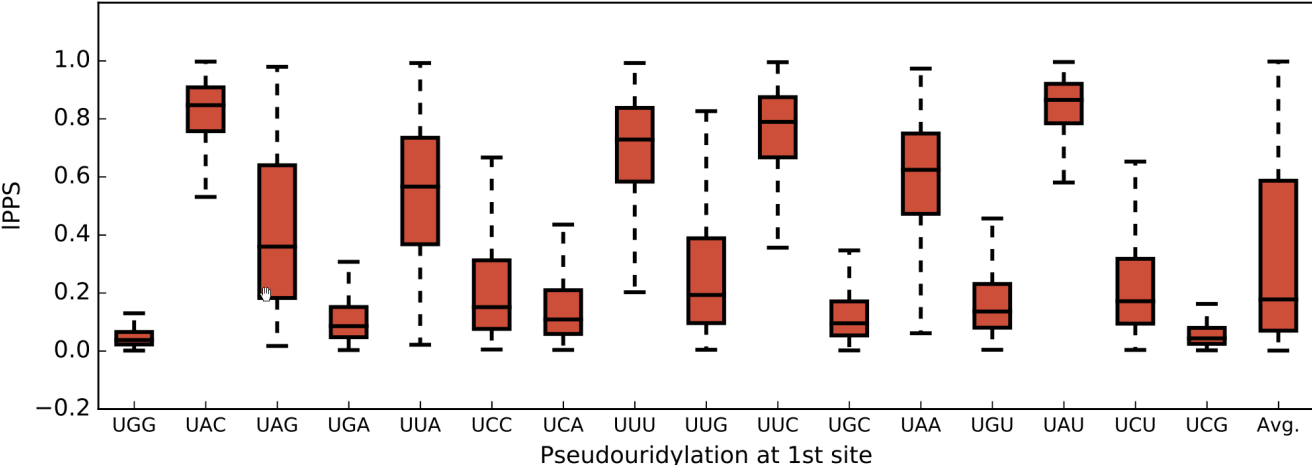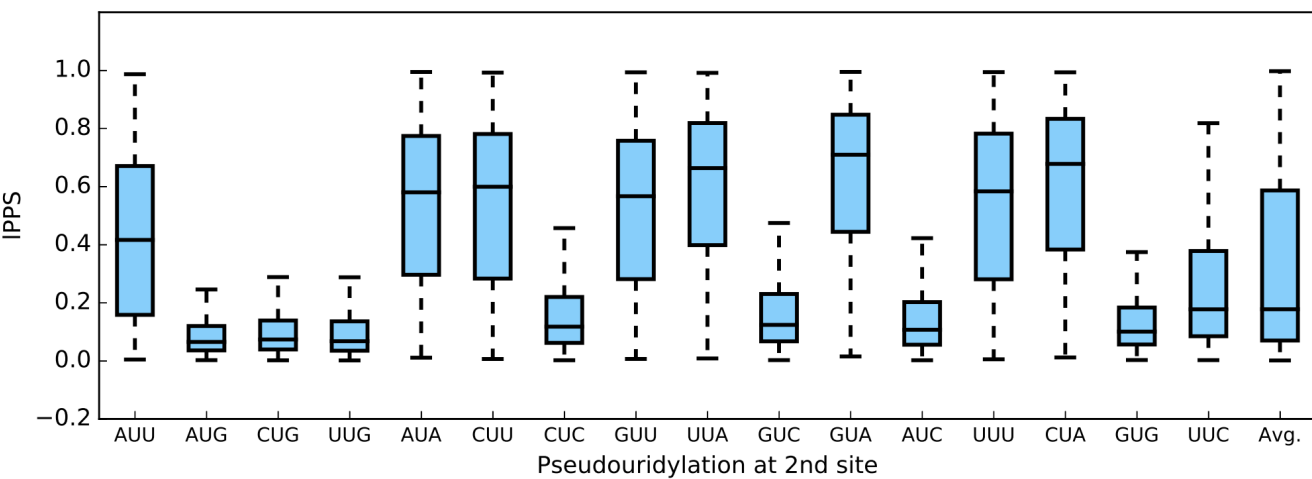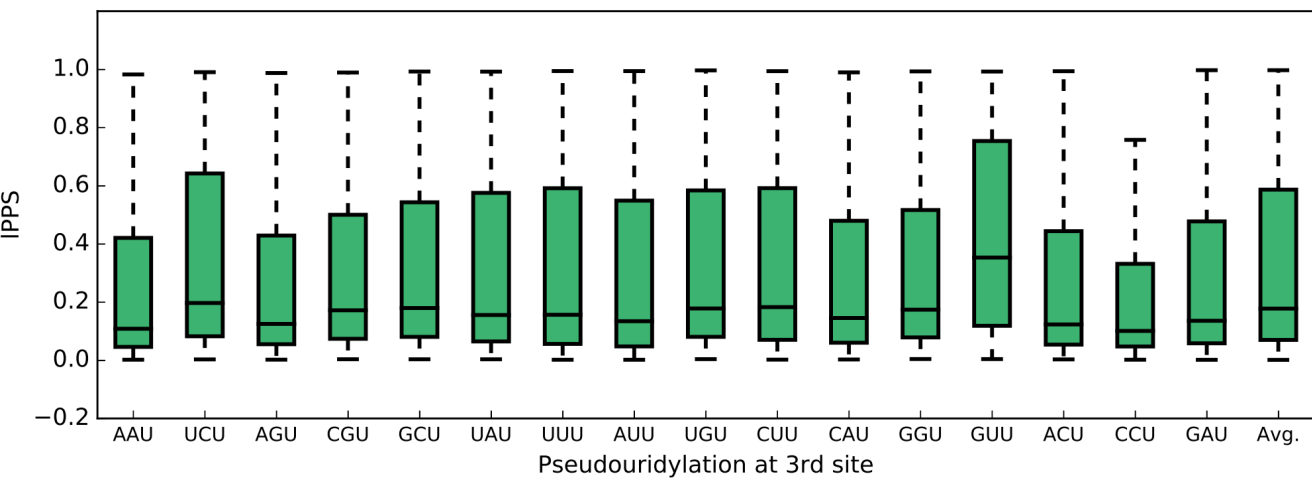

Supplement: Supplementary Figure S9 — Pseudouridine potentials of mouse uridine-containing codons with uridine in different nucleotide positions. The lPPS values predicted by PULSE for the uridine-containing codons with pseudouridylation in different nucleotide positions for mouse. The average lPPS value (termed by ‘Avg’) is also shown. [file mmc10.pdf]

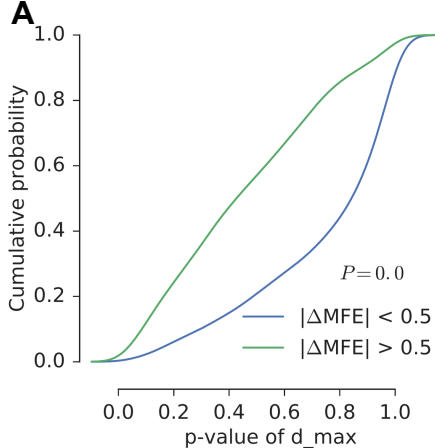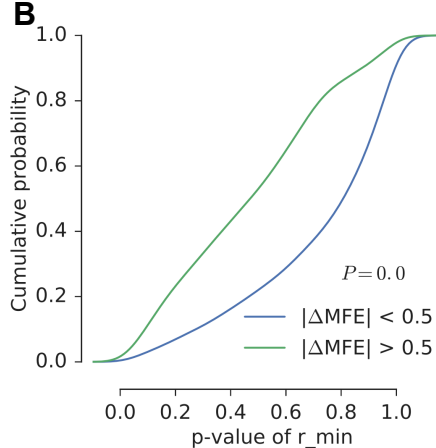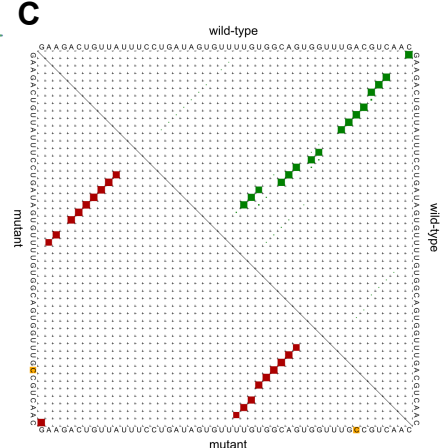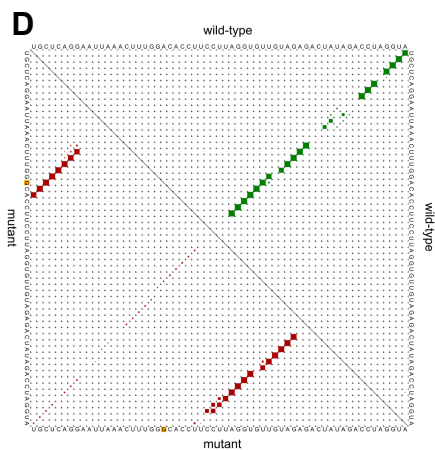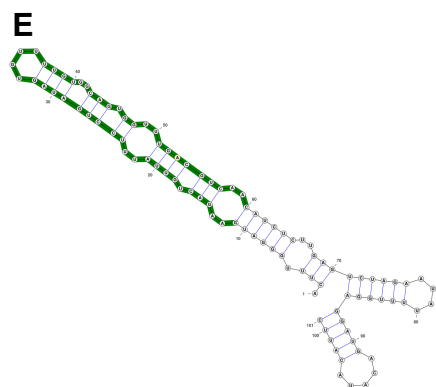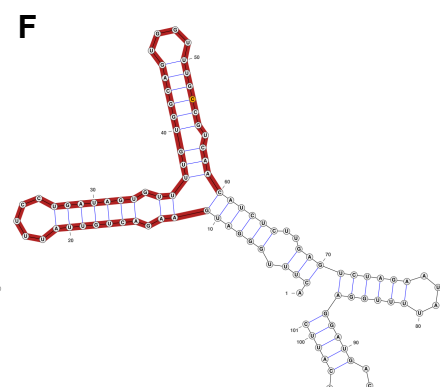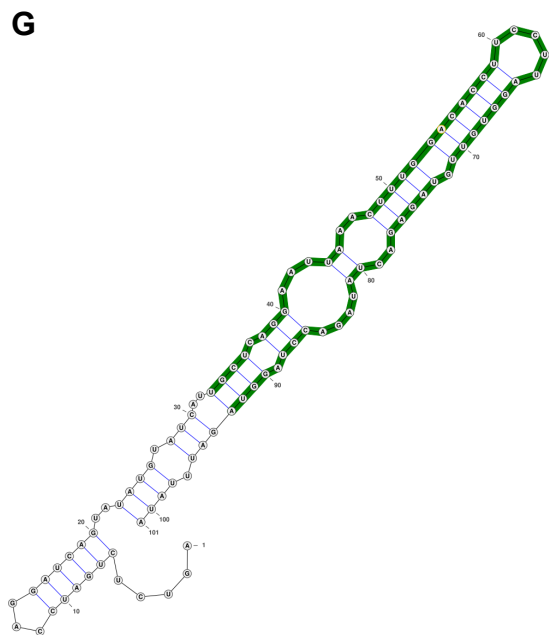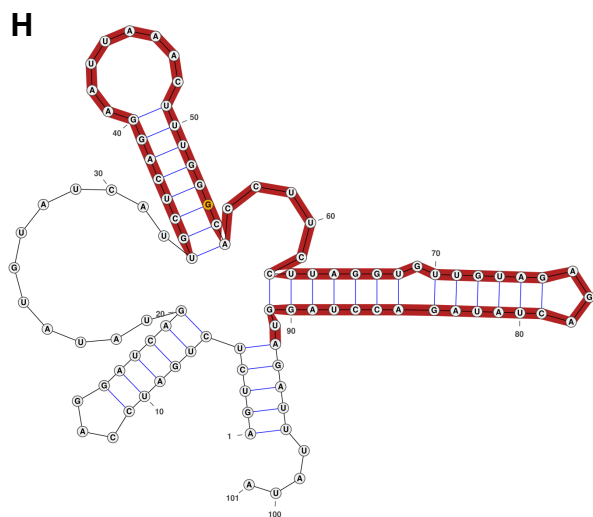

Supplement: Supplementary Figure S10 — The small change of free energy in a range of +/- 3 kCal/Mol can lead to a dramatic transformation of RNA secondary structure. A.–B. Cumulative curves of the p-values of the max base pairing distance (dmax) and the minimum coefficient (rmin) calculated by RNAsnp between the allele pairs with |ΔMFE| < 0.5 and |ΔMFE| > 0.5, respectively. C.–D. The dot plots of the RNA base pairing for two allele pairs. ΔMFE = 3.3 kCal/Mol (C);ΔMFE = 1.5 kCal/Mol (D). wild-type: major allele; mutant: minor allele. The mutant positions are highlighted in yellow. E.–F. The RNA structures corresponding to the wild-type and mutant alleles in (C), respectively. G.–H. The RNA structures corresponding to the wild-type and mutant allele in (D), respectively. [file mmc11.pdf]

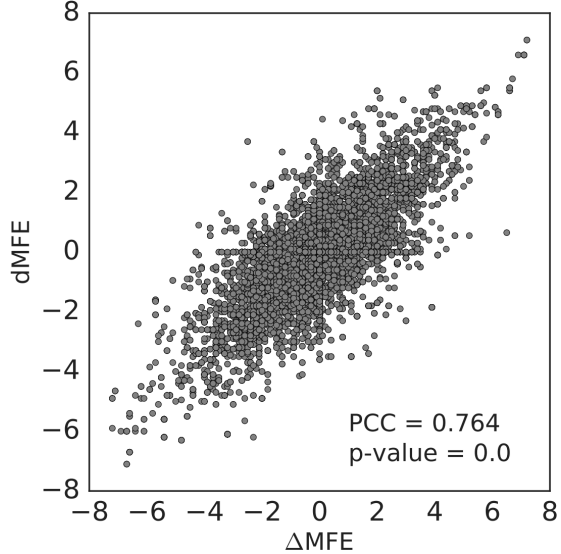

Supplement: Supplementary Figure S11 — Comparisons between ΔMFE and dMFE. The comparison between ΔMFE derived from our method and dMFE calculated by remuRNA. [file mmc12.pdf]

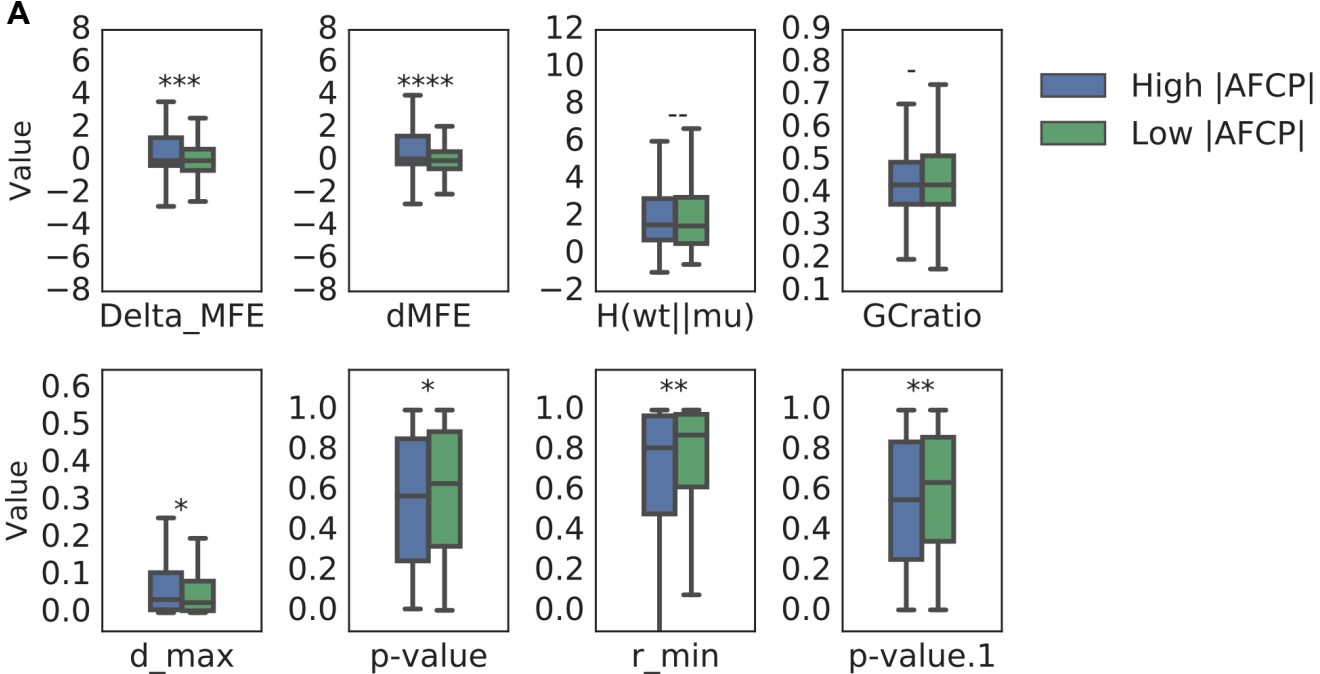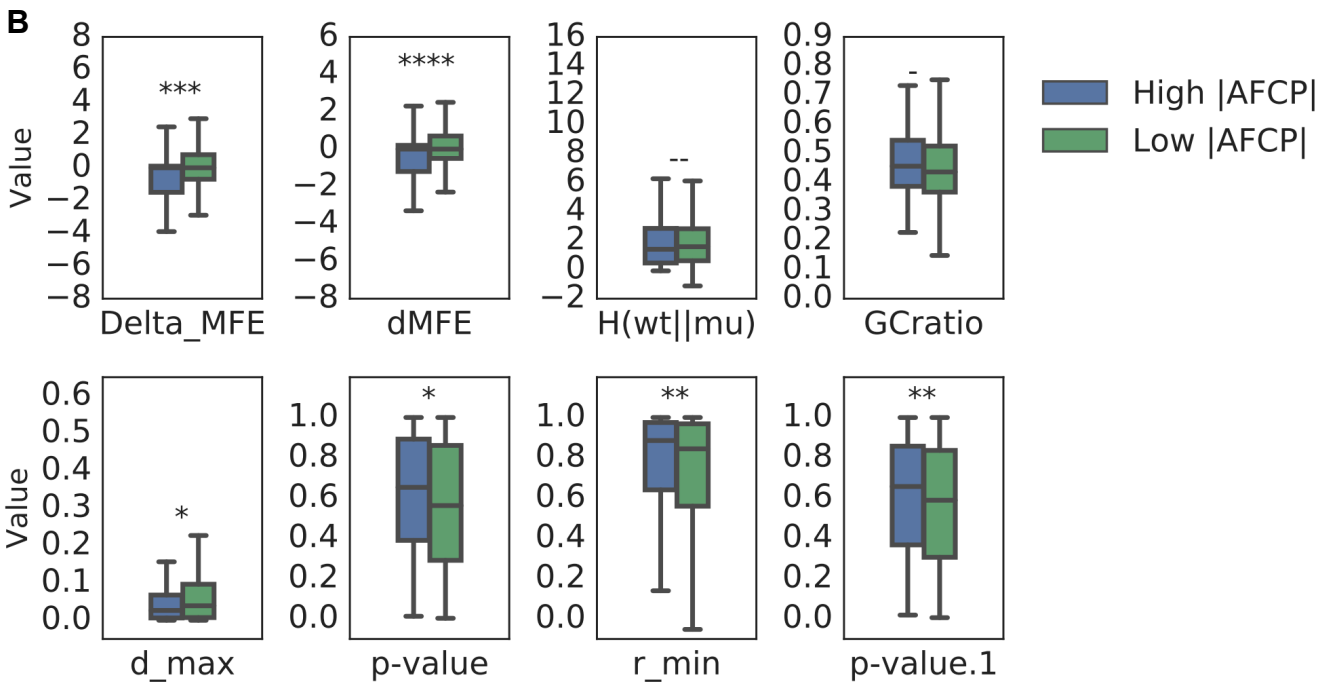

Supplement: Supplementary Figure S12 — Comparisons of the metrics derived by remuRNA and RNAsnp between high and low |AFCP| allele pair subgroups. A. Comparison results of the positive group (i.e., AFCP > 0.0). B. Comparison results of the negative group (i.e., AFCP < 0.0). For both positive and negative groups, samples were further divided into two subgroups, i.e., |AFCP| > 3 as high |AFCP| group and |AFCP| < 1.25 as low |AFCP| group. *, 10-5 < P < 0.01; **, 10-10 < P < 10-5; ***, 10-10 < P < 10-20; ****, P < 10-20; -, 0.01 < P < 0.1; --, P > 0.1; rank sum test for Delta_MFE, dMFE, P value and P value.1; Student's t test for the rest metrics. [file mmc13.pdf]
